# Copy number variation profile in the placental and parental genomes of recurrent pregnancy loss families

**DOI:** 10.1038/srep45327

**Published:** 2017-03-27

**Authors:** Laura Kasak, Kristiina Rull, Siim Sõber, Maris Laan

**Affiliations:** 1 Human Molecular Genetics Research Group, Institute of Molecular and Cell Biology, University of Tartu, Riia 23 St., Tartu 51010, Estonia; 2 Department of Obstetrics and Gynaecology, University of Tartu, Puusepa St. 8, Tartu 51014, Estonia; 3 Women’s Clinic of Tartu University Hospital, Puusepa St. 8, Tartu 51014, Estonia; 4 Institute of Biomedicine and Translational Medicine, University of Tartu, Ravila St. 19, Tartu 51014, Estonia

## Abstract

We have previously shown an extensive load of somatic copy number variations (CNVs) in the human placental genome with the highest fraction detected in normal term pregnancies. Hereby, we hypothesized that insufficient promotion of CNVs may impair placental development and lead to recurrent pregnancy loss (RPL). RPL affects ~3% of couples aiming at childbirth and idiopathic RPL represents ~50% of cases. We analysed placental and parental CNV profiles of idiopathic RPL trios (mother-father-placenta) and duos (mother-placenta). Consistent with the hypothesis, the placental genomes of RPL cases exhibited 2-fold less CNVs compared to uncomplicated 1^st^ trimester pregnancies (*P* = 0.02). This difference mainly arose from lower number of duplications. Overall, 1^st^ trimester control placentas shared only 5.3% of identified CNV regions with RPL cases, whereas the respective fraction with term placentas was 35.1% (*P* = 1.1 × 10^−9^). Disruption of the genes *NUP98* (embryonic stem cell development) and *MTRR* (folate metabolism) was detected exclusively in RPL placentas, potentially indicative to novel loci implicated in RPL. Interestingly, genes with higher overall expression were prone to deletions (>3-fold higher median expression compared to genes unaffected by CNVs, *P* = 6.69 × 10^−20^). Additionally, large pericentromeric and subtelomeric CNVs in parental genomes emerged as a risk factor for RPL.

Miscarriage is the most prevalent human gestational complication, mostly related to fetal chromosomal abnormalities^1^. Recurrent pregnancy loss (RPL) is a common disorder diagnosed based on ≥ 3 consecutive miscarriages before 22^nd^ gestational week^2^. It affects up to 3% of couples trying to conceive and has multiple etiologies, including maternal thrombophilic disorders and uterine abnormalities, immune and endocrine disturbances as well as gross cytogenetic aberrations in the parental karyotype. An abnormal embryonic karyotype has been reported for 41.4% of RPL cases^3^, possibly referring to systematic errors in meiotic and/or mitotic process in early development of the conceptus. Roughly 50% of the RPL cases remain unexplained^4^.

Indicative to the involvement of genetic factors, the prevalence of RPL among first-degree relatives of the idiopathic patients is increased 6-fold compared to the general population^5^. The challenge in investigating the inherited predisposition to RPL is summarized in the key question – ‘who is the case in recurrent miscarriage?’^6^ An ideal study would include the analysis of both, the genome of the parents as well as miscarried conceptus(es). Due to complexity and ethical issues in collecting clinical samples from RPL couples and pregnancy loss events, there are limited genetic studies on the RPL families.

Studies of structural genomic variants (also termed as copy number variations, CNVs, ranging from tens to millions of base pairs) in RPL cases are indicated. CNVs may affect the dosage of genes critical to early pregnancy or disturb normal chromosome segregation, possibly leading to aneuploidy. Currently, the reports on CNVs as potential risk factors to RPL are scarce. Our seminal analysis of CNV profiles in RPL couples identified two outliers out of 43 patients with an excessive (>5 Mb) cumulative burden of genomic rearrangements in their genomes^7^. This load of genomic rearrangements possibly increases the risk of meiotic errors and of impaired chromosome segregation during rapid cellular division and differentiation after fertilization. The study also discovered a specific multicopy duplication (61.6 kb) at 5p13.3 conferring increased maternal risk to RPL. Regarding the CNV profile of the miscarried conceptuses from RPL cases, there is only one published study using low-resolution aCGH approach and identifying a number of CNVs specific to miscarriage events^8^. Two maternally inherited rearrangements included imprinted genes (*CTNNA3* and *TIMP2*) that regulate trophoblast invasion and are normally expressed from only the maternal allele in the placenta. So far, there is no clear understanding whether and to what extent the combined effect of CNV profiles in the genomes of RPL couples and their miscarried conceptus(es) may predispose to consecutive pregnancy losses.

Recently, we published the first comparative analysis of CNVs in the parental and placental genomes across all three trimesters of human pregnancy. As a major outcome, we reported an extensive load of somatic CNVs, especially duplications in the placental genome^9^. As the highest number of placental CNVs was detected for healthy term pregnancies, we suggested that this phenomenon might be critical for normal gestation. Similarly to cancerous cells, placental somatic rearrangements may have a role in promoting rapid cellular proliferation, migration and deep trophoblast invasion within a short and critical timeframe. In the current study, we hypothesized that the placental genomes of recurrent pregnancy loss cases are characterized by insufficient promotion of genomic rearrangements, which may impair early placental development and establishment of a viable pregnancy. In addition, we assessed the potential contribution of specific risk CNVs identified in either parental or placental genomes of RPL cases, harbouring genes critical for early development. This is the first report profiling CNVs in the genomes of RPL family trios (mother-father-placenta) and duos (mother-placenta) in comparison to the parental-placental control samples representing uncomplicated early and term pregnancies.

## Results

### RPL cases have significantly less placental CNVs compared to normal pregnancy

**Figure 1:**
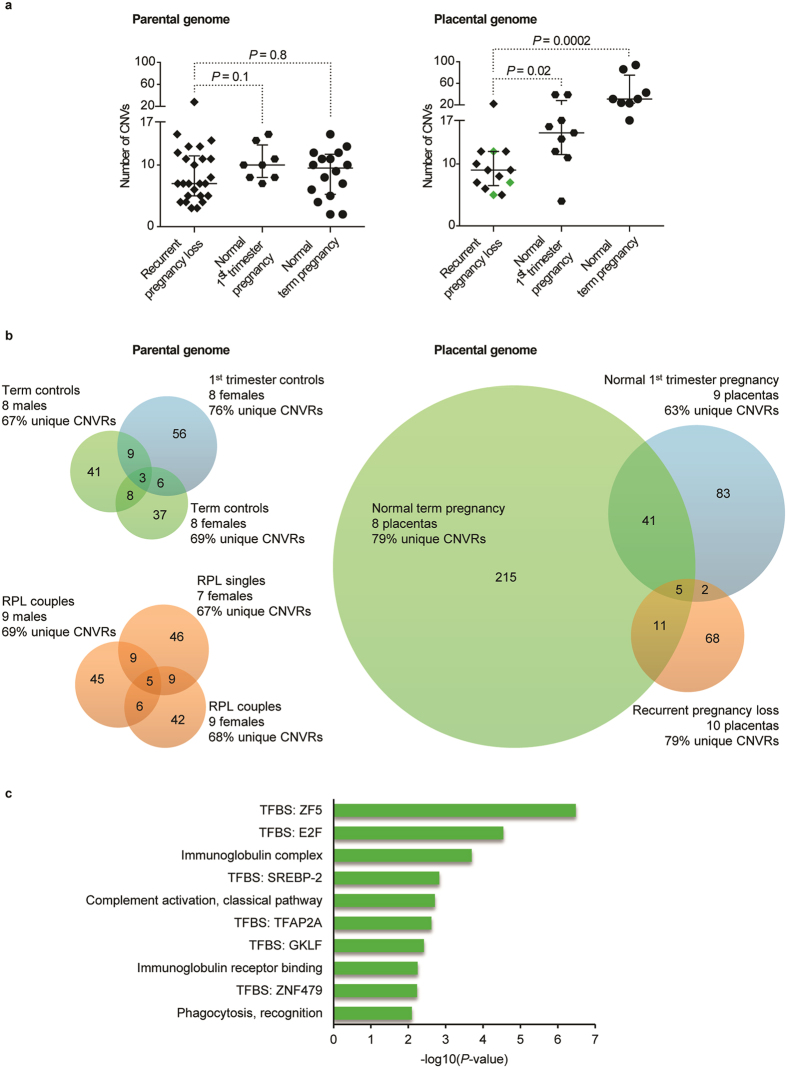
Comparison of autosomal CNVs in the placental and parental samples of recurrent pregnancy loss (RPL) cases compared to controls representing normal 1^st^ trimester and term pregnancy. (**a**) Parental genomes of RPL patients and controls show no significant difference in the number of CNVs. A significantly lower number of CNVs was detected in the placental genomes of RPL patients compared to normal pregnancies; placentas from RPL couples eventually succeeding in a term live birth after a successful pregnancy are indicated with green diamonds. *P*-values for the differences among the groups were calculated by Wilcoxon rank sum test. Error bars show median values with interquartile range. (**b**) Venn-diagrams illustrating the degree of overlap in CNVRs detected in the parental and placental genomes from RPL cases and controls. (**c**) Gene enrichment analysis of CNVRs exclusively detected in the placental genomes of 1^st^ trimester and term control pregnancies. TFBS, transcription factor binding site.

Whole-genome profiling of duplications and deletions in the parental blood and placental samples was carried out using a SNP array based CNV calling (>713,000 markers with mean spacing 4.0 kb). There was no statistical difference for the number of duplications and deletions and for the cumulative span of all CNVs in the parental genomes of recurrent pregnancy loss (RPL) patients and controls (Fig. 1a; Table 1, Supplementary Table S1).

In contrast to parental blood DNA samples, the placental genomes exhibited substantial differences. Placental genomes of RPL cases exhibited 40% reduction in the number of CNVs compared to normal 1^st^ trimester pregnancies (median number of CNVs/genome: 9 *vs.* 15; Wilcoxon rank sum test, *P* = 0.02; Fig. 1a; Table 1). When comparing the RPL cases with term pregnancies, there was a 71% decrease (9 *vs.* 31; *P* = 2.46 × 10^−4^; Fig. 1a; Table 1).

The difference in the load of CNVs was mainly due to 3–10 fold reduction in duplications in the placentas of RPL patients compared to controls (Fig. 1a, Supplementary Fig. S1). Whereas the number of duplications in RPL placentas (median: 2.5 for miscarriage and 3 for live birth placentas) was comparable with the parental genome estimates (n = 2), placentas from uncomplicated early and term pregnancies exhibited median 8.0 and 25.5 duplications, respectively (*P* = 5.28 × 10^−4^; Table 1). Consequently, the cumulative span of placental CNVs for the controls (1^st^ trimester, median 1.8 Mb; term 4.4 Mb) was manyfold greater and statistically different from the pregnancies of RPL couples, irrespective of their outcome (miscarriage, 0.6 Mb; live birth, 0.9 Mb; *P* = 1.04 × 10^−3^).

We have previously shown that the high load of CNVs in the human placental genome arises mainly from somatic duplications^9^. Notably, the seminal study also demonstrated that placentas from late pregnancy complications such as preeclampsia, gestational diabetes and fetal growth disturbances exhibit significantly lower number of CNVs. Among term complications the lowest average number of placental CNVs was detected in the small-for-gestational age (SGA) group; however, in the RPL placentas it was almost 2-fold further reduced (18.3 *vs.* 10.0 CNVs, respectively; Supplementary Fig. S1). This suggests a general feature to all pregnancy complications – a reduced capacity to promote somatic genomic rearrangements in the placental genome. However, this appears to be more extreme in RPL pregnancies.

### Low fraction of shared CNVs in the placental genomes of RPL and normal pregnancies

Next, we clustered CNVs into CNV regions (CNVR) and assessed their genomic distribution and content in the placental genomes from RPL (n = 10, Supplementary Table S2) compared to normal 1^st^ trimester (n = 9) and term pregnancies (n = 8). The total pool of CNVRs was the smallest in the genomes of miscarried placentas (n = 86; 8.6 per sample), followed by normal 1^st^ trimester (n = 131; 14.6 per sample) and term pregnancy placental samples (n = 272; 34 per sample). None of the groups stood out for the overall ratio of unique to shared CNVRs (63–79%; Fig. 1b), which fell in the expected range when three similar-sized groups of unrelated genomes were compared (parental blood DNA, 69–76% unique CNVs; Fig. 1b). Four of the five placental CNVRs (exception: the *MTRR* region, *see below*) shared by all groups were located in intergenic regions or known structurally polymorphic loci (e.g. *alpha-Amylase* and *Pregnancy-specific Glycoprotein (PSG*) gene clusters). It is noteworthy that normal 1^st^ trimester samples shared 35.1% (n = 46/131) of CNVRs with term placentas, but only 5.3% (n = 7/131) with RPL cases (Fisher’s exact test, *P* = 1.1 × 10^−9^).

**Figure 2:**
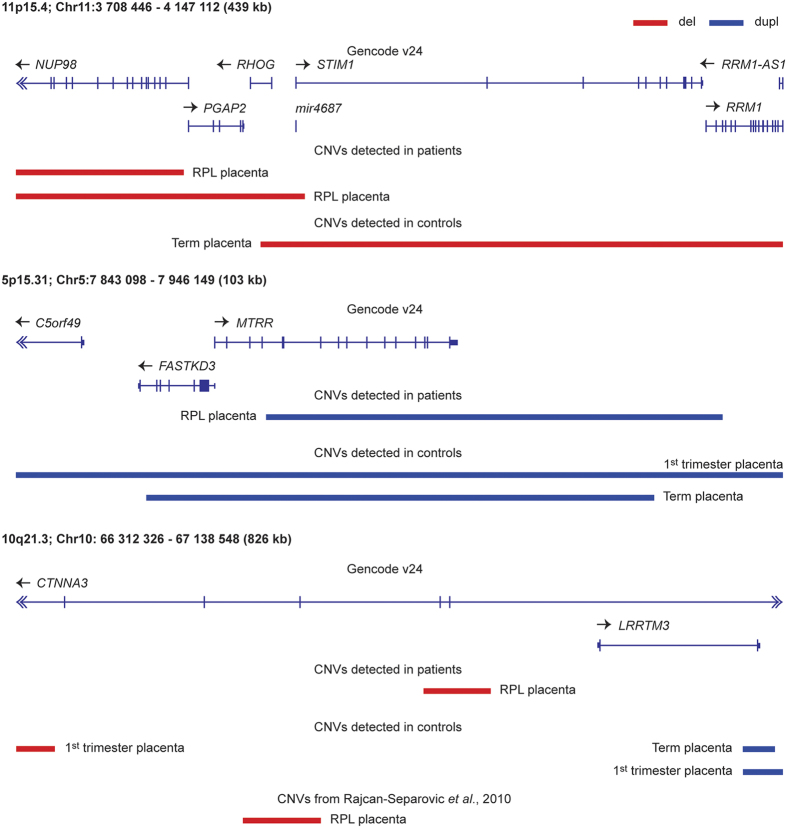
Genomic context of three alternatively rearranged regions in the pregnancy loss (RPL) compared to normal 1^st^ trimester and term pregnancy placentas. Blue bars indicate duplication (dupl) and red bars deletion (del) CNVs.

Five of the 18 CNVRs shared between the control and RPL samples (Fig. 1b), and three additional genomic regions with rearrangements coinciding within the same gene, harboured alternative, possibly gene function affecting CNVs in miscarried placentas (Table 2). For example, overlapping deletions at 11p15.4 were detected for one normal term and two RPL placentas. However, only the latter two involved the gene *NUP9*8 encoding a nuclear pore protein (Fig. 2). NUP98 interacts with the human genome in a dynamic manner that is tightly linked to the developmental stage and has a role in gene regulation during human embryonic stem cell differentiation^10^. NUP98 is also involved in chromosomal translocations (involving ~30 different genes) in a subset of acute myeloid leukemia patients^11^. Two control placental genomes had duplications involving the entire *MTRR* (methionine synthase reductase) gene (5p15.31), but the duplication carried by an RPL placenta disrupted the gene (Fig. 2). *MTRR*, encoding an enzyme involved in folate metabolism, has been associated with preeclampsia^12^, spontaneous abortion^13^ and idiopathic RPL^14^. Although all the identified CNVs at 10q21.3 encompassed an imprinted and maternally expressed gene *CTNNA3*, the rearrangements in three control placentas were distinct from the maternally inherited deletion (57.1 kb) detected in the RPL placental genome (Table 2). Importantly, a maternally transmitted deletion at the same region has been reported in an independent study on recurrent miscarriage samples^8^. Both RPL group specific CNVs involved different exonic parts of *CTNNA3* gene while the rearrangements in control placentas covered only intergenic regions (Fig. 2).

Small sample size and mostly singleton placental samples restricted the analysis of CNVs that may predispose to RPL in individual families. Only two mothers had placental samples available from two separate miscarried pregnancies. Placental samples from the RPL89 family shared a maternally inherited 80 kb deletion involving the *MSR1* gene that encodes the class A macrophage scavenger receptors, and a 200 kb duplication encompassing two genes, *DOCK8* and *KANK1*. A *de novo* distal 9p deletion involving the same genes has been associated with abnormal maternal serum screening result and intrauterine growth restriction^15^. The two miscarried RPL71 placentas shared a 250 kb deletion involving the *LRP5L* gene with high expression in female reproductive tissues.

### Gene enrichment analysis of placental CNVRs specific to RPL cases and controls

Functional profiling of genes located within the CNVRs identified exclusively in control placental samples highlighted an enrichment of binding sites for several transcription factors (TF) (Fig. 1c, Supplementary Table S3). For 81% and 71.3% of the query genes (n = 630) a binding motif for the ZF5 (*P* = 3.27 × 10^−7^) and E2F (*P* = 2.86 × 10^−5^) transcription factors was detected. E2Fs regulate the process of endoreplication, characteristic to trophoblastic cells, and coordinate the placental transcriptional network to guarantee proper placental development and fetal viability^16^. Another highlighted factor, TFAP2A (*alias* AP2α; *P* = 2.38 × 10^−3^; 16.8% of query genes) regulates the expression of hCG beta genes^17^, encoding a placental hormone critical in early gestation^18^. A syncytiotrophoblast marker gene *GKLF (alias KLF4*) has been shown to be involved in impaired trophoblastic differentiation and implantation failure in unbalanced t(11;22) embryos^19^. The detected phagocytosis and immune function related biological pathways are also relevant in pregnancy maintenance. On the other hand, analysis of CNVRs unique to RPL placentas showed an enrichment of only highly polymorphic olfactory receptor genes (Supplementary Table S3).

### Pilot analysis of expression levels of genes in loci affected by CNVs

**Figure 3:**
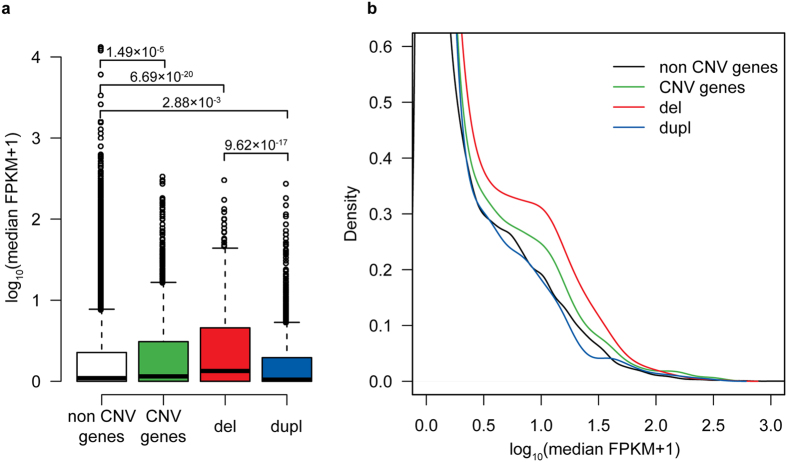
Profiles of median expression values of genes overlapping with CNVs (‘CNV genes’; n = 2,273) compared to genes not overlapping with CNVs (‘non CNV genes’; n = 45,156) in all placental samples with RNA-Seq data^20^,^21^ (n = 50; RNA-Seq dataset details in ref. 21 and Supplementary Methods). Expression levels are measured as FPKM (fragments per kilobase per million) and distributions are shown as boxplots (**a**) and plotted density estimates with Gaussian kernel (**b**). Genes affected only by deletion CNVs (del; n = 1148) and duplication CNVs (dupl; n = 1063) are shown separately. *P*-values for between-group comparisons are derived from Wilcoxon rank sum tests.

For 50 placental samples profiled for their CNV content in the current and previous study^9^, also RNA-Seq dataset has been generated^20^,^21^. In order to assess the functional effect of CNVs on the expression of the involved genes (termed as ‘CNV genes’; n = 2,273 across all samples), we compared their transcript levels with the remaining Ensembl genes, not disrupted by CNVs in any placental samples (‘non CNV genes’; n = 45,156). Across all samples, ‘CNV genes’ had > 1.5-fold higher expression level compared to ‘non CNV genes’ (median FPKM (Fragments Per Kilobase of transcript per Million mapped reads) = 0.151 *vs.* 0.094, respectively; *P* = 1.49 × 10^−5^; Fig. 3a, Supplementary Table S4). Interestingly, the expression level of genes within genomic regions prone to deletions (n = 1,148) was even higher (median FPKM = 0.341; *P* = 6.69 × 10^−20^). However, the overall profile of median expression values of genes within genomic regions involved in duplications (n = 1,063) was similar to the ‘non CNV gene’ group (Fig. 3b) and even showed a tendency towards decreased transcript levels (median FPKM = 0.049; *P* = 2.88 × 10^−3^; Fig. 3a, Supplementary Table S4). In order to exclude gestational age as the potential confounding factor, identical analysis was also performed for only term placental samples (n = 40, RNA-Seq data from ref. 20). The outcome of this analysis was concordant with the results obtained for the whole dataset (Fig. 3, Supplementary Fig. S2, Supplementary Table S4).

To analyse individual level effects of CNVs on gene expression, DESeq2 normalized (default settings) read counts of ‘CNV genes’ were transformed to z-scores and compared between individuals with deletions, duplications and without CNVs. This analysis did not reveal statistically significant consistent gross effects of deletions or duplications on the expression levels of involved genes (Wilcoxon rank sum test *P* values > 0.05, data not shown). However, the analysis had limited power as the number of carriers of each CNV was low and the vast majority represented singleton variants.

### Large parental pericentromeric and subtelomeric CNVs may predispose to RPL

Parental genomes of RPL cases exhibited almost twofold excess of >300 kb CNVs compared to controls (8.6 *vs.* 4.1% of all CNVs, *P* = 0.08; Table 3, Supplementary Fig. S3a,b). Notably, 63% of these large CNVs present in RPL were mapped to pericentromeric and subtelomeric regions, compared to only 33% in the control parental genomes (12 *vs.* 3 of all > 300 kb CNVs; Table 3, Supplementary Fig. S3).

A male partner of the couple RPL7 was detected to carry a 0.5 Mb pericentromeric duplication at 15q11.2, not identified by a conventional karyotype analysis (hg38: Chr15:22,584,820 – 23,122,762; Supplementary Fig. S3c). The couple had experienced in total 6 pregnancy losses. The identified large CNV is located within a known 15q11.2-13 microdeletion/duplication syndrome region (13 Mb; OMIM:608636), implicated in Prader-Willi and Angelman syndromes. The 500 kb duplication resides between the established rearrangement breakpoints (BP1, BP2)^22^,^23^ at the edge of the core microdeletion/duplication region.

Among other large CNVs, two patients (RPL11 female; RPL45 male partner) carried rearrangements encompassing genes from Ubiquitin-specific peptidase family, *USP10* and *USP25*, which are related to DNA damage response^24^ and aneuploidy syndromes^25^. Other members of the Ubiquitin-specific peptidase family have in fact been associated with male infertility and also RPL^26^.

### Parental and placental CNV profile in the RPL couples succeeding in live birth

In two of the three RPL families with placental samples representing live births at term, either one (RPL3 father) or both of the parents (RPL11) carried large pericentromeric CNVs (0.3–0.6 Mb/genome; Supplementary Table S5). RPL11 mother also carried one large subtelomeric rearrangement. However, these CNVs have not been transmitted to the successful pregnancies. Furthermore, in live birth placental samples from RPL couples only somatic CNVs (RPL11, RPL12) or paternally inherited CNVs were detected (RPL3). The RPL3 mother has a deletion on Chr20 encompassing the *MACROD2* gene that has been associated with antiphospholipid syndrome (APS)^27^. APS represents an acknowledged high risk factor for RPL^1^. Notably, although the total number of placental CNVs in RPL live birth cases was as low as in miscarried pregnancies, these regions encompassed genes duplicated also in term control samples. There was 42% overlap in respective gene content compared to only 25% in miscarriage placentas, including e.g. *ADRA2C* that is highly expressed in the endometrium, uterus and also placenta. This may have supported the successful establishment and development of these index pregnancies.

As an interesting observation, the babies born to the RPL11 (male 4,488 g) and RPL12 (female 5,060 g) couples were large-for-gestational age (LGA) (Supplementary Data S1). Both couples were diagnosed with primary RPL cases, had further pregnancy losses after the index case and another successful pregnancy resulting again in LGA cases (RPL11: male 4,990 g; RPL12: female 4,590 g). This suggests that there might be compensatory mechanisms (e.g. through epigenetic programming) in the early placental development that enable to overcome the deficiencies in the placental genome and to maintain the pregnancy. However, this may lead to placental malfunction and in turn, to fetal overgrowth.

## Discussion

Our previous study revealed a load of somatic CNVs, especially duplications, in the placental genomes of successful pregnancies^9^. CNVs enriched for genes involved in immune regulation, cell adhesion, embryonic development and cell cycle may modulate the expression of relevant genes at specific time points to guarantee normal progression and maintenance of pregnancy. Supportively, others have reported selective amplification of genomic regions in mouse trophoblast giant cells containing placental gene families with essential role in murine pregnancy (prolactins, serpins, cathepsins)^28^.

The current study explored the hypothesis that impaired promotion of placental genome rearrangements during early pregnancy may represent an unacknowledged risk factor for recurrent pregnancy loss (RPL). As a major finding, we report more than 40% reduction in the number of CNVs detected in the placental genomes of RPL cases compared to uncomplicated pregnancies (Table 1, Fig. 1a). As the overall number of CNVs in the placental genomes of RPL cases was in the same range as the parental genomes, it supports the idea of impaired promotion of somatic structural variation in placentas as a contributor to pregnancy loss.

We detected disruption of *MTRR* (methionine synthase reductase) and *NUP98* genes in RPL placental genomes but not in controls (Fig. 2, Table 2). *MTRR* is essential for utilization of methyl groups from the folate cycle, required for the methylation of DNA and histones. Disrupting a key enzyme involved in folate metabolism may have important implications on epigenetic dysregulation of many genes and entire pathways. Especially in early embryonic and placental development, folates are crucial to support the proper gestational dynamics of the genome during the intrauterine period^29^. In mice, *Mtrr* deficiency has been shown to have transgenerational effect *via* epigenetic markers, causing growth defects and congenital malformations^30^. Furthermore, *MTRR* 66 A > G polymorphism (rs1801394) has been associated previously with idiopathic RPL and pregnancy complications^13^,^14^. *NUP98*, on the other hand, is a novel gene implicated in miscarriages. In human embryonic development, *NUP98* is involved in regulating genes responsible for cell cycle and nucleic acid metabolism^10^. Additionally, this gene has been linked with whole chromosome instabilities, mitotic spindle defects and chromosome mis-segregation^31^. Another disrupted region represented a deletion encompassing *CTNNA3* gene, detected in recurrent miscarriage samples in an independent study previously^8^. *CTNNA3* encodes αT-catenin, which is a cell-adhesion molecule that regulates the balance between proliferation and invasion of trophoblasts^32^.

CNVRs exclusively present in 1^st^ and 3^rd^ trimester control placental samples showed enrichment for the binding sites of several transcription factors (TFs) known to be involved in placental development and function (Fig. 1c, Supplementary Table S3). Among these, members of the *E2F* transcription factor family are known to orchestrate mammalian endoreplication process^16^. The latter is an essential part of mammalian extraembryonic tissue function. Consistently, a study on the placental transcriptome in RPL cases has identified *E2F* TF-family as a potential key to trigger the fetal programming towards pregnancy loss^21^. Consistent with the critical role in the placental gene expression, *E2F* as well as other TFs highlighted in the current study (*ZF5, GKLF alias KLF4, AP2, SREBP*; Fig. 1c) have been shown to deregulate the transcriptional landscape in preeclamptic (PE) placentas^20^,^33^. RPL and PE have both been linked to impaired early placental development and have previously been demonstrated to share similar etiological factors^7^,^34^. Rearrangements of genes acting as transcriptional regulators for the cell cycle process and trophoblast proliferation suggests an alternative mechanism to repress or activate genes at specific time points during placental/embryonic development.

Our pilot analysis of the expression of genes involved in placental CNV regions (CNVR) resulted in unexpected outcomes. Genes located within identified CNVRs showed a higher expression level compared to those not disrupted by CNVs in any placental genomes (Fig. 3). Especially genes located within the regions prone to deletions showed a three-fold higher median expression level compared to the remaining gene pool, whereas genes located in loci harbouring duplications exhibited a tendency to lower expression. The mechanisms behind these observations and their functional consequences are still to be discovered. This study was not adequately powered to identify statistically significant gross effects of CNVs on the level of individual genes. Analysis of individual loci is hampered by low frequency of the majority of CNVs, as well as possibly by placental somatic mosaicism. Also, the current study design did not allow proper adjustment for the gene expression confounding factors in each comparison of carriers and non-carriers of a particular CNV. Further targeted studies are needed for the assessment of the effect of individual CNVs on modulating the function of the placental genome. For example, a recent study on cancer genomes showed that somatic CNVs also modify expression of nearby genes by re-adjusting genomic structure and location of regulatory elements^35^.

Parental genomes of RPL cases revealed almost twofold increase in the proportion of large (>300 kb) CNVs compared to controls, preferentially mapped to pericentromeric or subtelomeric regions (Supplementary Fig. S3). Consistently, acrocentric pericentromeric and also subtelomeric abnormalities have been associated with RPL previously^36^. Clinical history analysis of our patients (Supplementary Data S1) also revealed multiple malformations in their previous pregnancy losses as well as in at least one newborn, indicative to possible disturbances in chromosomal segregation in early development. In our study, one male partner carried a 500 kb pericentromeric duplication at 15q11.2, located within a known microdeletion/duplication region. In the literature, the phenotypic consequence of this smaller rearrangement is currently not fully established. Although 40% of the 15q11.2 BP1-BP2 microduplication carriers suffer from delayed development and speech, autism and other neuro-behavioural problems, phenotypically normal carriers have been identified in several instances, complicating phenotypic association and/or causality^22^,^37^. Supportively to this study, CNVs at the 15q11 genomic region were recently reported as a common finding for the cases of spontaneous 1^st^ trimester euploid miscarriages (37.8% of analysed samples)^38^. Large genomic rearrangements in RPL parental genomes are expected to increase genomic instability. Substantial rearrangements in the pericentromeric and subtelomeric regions could interfere with correct chromosome pairing and segregation in meiosis and mitosis, thereby making their genomes ‘unfavourable’ to produce a viable offspring. Consistent with this idea, none of the RPL live birth placental samples had inherited large subtelomeric or pericentromeric CNVs present in their parental genomes. The birth of large-for-gestational age babies in couples with primary RPL may refer to impaired placental function due to altered programming of the placental genome in order to guarantee compensatory mechanisms for the support of the fetal development, survival and growth.

We acknowledge a small sample size as a limitation in the current study. Nevertheless, our research results may have a number of perspective implications. Firstly, uncovering of a novel potential predisposing factor for RPL promotes future studies to identify the molecular mechanism behind initiation of genomic rearrangements in early placental genome. So far, there is not enough scientific data to speculate on the exact mechanism behind placental somatic CNVs and failure to properly promote these in RPL cases. Genomic rearrangements might be regulated through differential local epigenetic environments, relaxed DNA repair mechanisms resembling the generation of somatic rearrangements in cancer genomes, (polymorphic) clusters of repetitive elements etc. In turn, the somatic CNVs can also modify local epigenome, long-range gene regulatory interactions and consequently, their expression. Uncovering the role of specific transcription factors (*E2F, AP2, KLF4* and *ZF5*) involved in this process in forthcoming studies may lead to new targets of treatment and management for RPL. Secondly, as we have previously briefly discussed the resemblance of placenta and tumour tissue^9^, novel insights in halting the process of genome rearrangements could potentially benefit the cancer research. Thirdly, the enrichment of large subtelomeric and pericentromeric CNVs in the parental genomes of RPL couples encourages the managing clinicians to opt for microarray-based analysis of microdeletions/duplications, additionally to conventional cytogenetics. Identification of an ‘unfavourable’ genome in one or both of the partners would allow improved counselling of the couples about the risks related to pregnancy and newborn health, and choosing the appropriate clinical management. Counselling in these cases could be considered similar to the couples with parental balanced chromosomal translocations. There is also no cure for this condition, but the patients are diagnosed for the cause of the pregnancy loss and made aware that there is a high possibility that next time they may succeed with the birth of a completely healthy child. According to clinical recommendations, RPL couples are explained that every conceptus carries a unique combination of genetic material from both partners and the next pregnancy may be successful without pharmacological intervention if offered counselling and supportive care^39^. In addition, our study outcome alerts to analyse placental material of miscarried conceptuses not only for gross chromosomal abnormalities, but also for the entire profile of CNVs to identify an ‘unfavourable’ placental genome as the possible cause for a pregnancy loss and to provide respective counselling.

## Methods

### Ethics statements

The study was approved by the Ethics Review Committee of Human Research of the University of Tartu, Estonia (permissions no 117/9, 16.06.2003; 146/18, 27.02.2006; 150/33, 19.06.2006; 212/M-32, 09.03.2012) and was carried out in compliance with the Helsinki Declaration. A written informed consent to participate in the study was obtained from each individual prior to recruitment. All study participants were recruited and the study material was collected at the Women’s Clinic of Tartu University Hospital and Nova Vita Clinic, Tallinn, Estonia 2003–2012. All participants were of white European ancestry and living in Estonia.

### Study groups representing RPL

The analysed idiopathic RPL patient group comprised couples (n = 9; female and male partners aged 23–38 and 24–40 years, respectively) and female patients (n = 7; 25–42 years; DNA samples of the male partners were unavailable). Unexplained RPL had been diagnosed using the following criteria: ≥ 3 consecutive pregnancy losses before week 22 of gestation without any identified cause (Table 4). Generally acknowledged clinical risk factors of RPL had been excluded (see below). Placental samples could be collected for a subset of miscarried index pregnancies of the recruited RPL cases (n = 10 (7 XX, 3 XY); 42–130 gestational days (g. d.)). Three of the analysed RPL couples had finally succeeded to reach a viable pregnancy and a live birth after a series of miscarriages; the respective term (1 boy, 2 girls; 284–287 g. d.) placental samples were included into the analysis as a comparative sample-set for miscarried gestations. Clinical details of each analysed RPL family or female case (age, BMI, total number of pregnancies, clinical history of pregnancy losses/live births/elective abortions etc.) are provided in Supplementary Data S1. In total, the analysed RPL dataset comprised 25 parental and 13 placental samples (Table 4). Parental blood DNA samples of four RPL couples (RPL3, RPL11, RP12, RPL45) and two female patients (RPL7, RPL63) overlapped with our previous CNV profiling study^7^ using low resolution SNP arrays (Illumina Human370CNV-Quad; 370,000 markers). In order to enable uniform CNV calling across all samples in the current study, these samples were re-genotyped using a denser SNP array (Illumina HumanOmniExpress-24-v1 BeadChips; > 713,000 markers) (Supplementary Methods).

Normal karyotype was confirmed for all RPL patients based on the DNA extracted from peripheral blood samples. Female RPL patients had normal regular menstrual cycles (mean 28 ± 3 days), no major uterine anomalies (based on ultrasonography or hystero-sonogram), exclusion of antiphospholipid syndrome and thrombophilic mutations (*FV* [MIM 612309] Leiden, p.Arg506Gln, rs6025^40^; *F2* [MIM 176930], c.G20210A, rs1799963^41^). Patients carrying the identified maternal risk CNV for RPL among Estonians and Danish^7^ – a 61.6 kb *GOLPH3-PDZD2* multicopy duplication at 5p13.3, were not included into the study.

### Study material representing uncomplicated 1^st^ trimester pregnancy

The dataset utilized in the current study representing normal 1^st^ trimester uncomplicated pregnancy is derived from our previous report^9^. Placental (n = 9; 5 XX, 4 XY) and maternal blood samples (n = 8) were obtained from women (aged 18–33 years), who underwent elective surgical termination of pregnancy during the 1^st^ trimester of gestation (ETP group; 51–81 g. d.; Table 4). Details for this patient group are provided in Supplementary Data S1.

### Study material representing uncomplicated term pregnancies

The dataset utilized in the current study representing normal term pregnancy is derived from our previous report^9^. Term pregnancy cases had been selected from the REPROgrammed fetal and/or maternal METAbolism (REPROMETA) study. REPROMETA families had been recruited shortly prior to term delivery. The collected study material includes clinical and epidemiological data and biological samples from normal singleton pregnancies at term (260–291 g. d). The biological sampling included placenta-mother-father trios (n = 8 (5 boys, 3 girls); female and male partners aged 18–37 and 22–38 years, respectively; Table 4). Cases with documented fetal anomalies, chromosomal abnormalities, families with history of inherited diseases and patients with known pre-existing diabetes mellitus, chronic hypertension and chronic renal disease were excluded. Detailed characteristics of REPROMETA samples included into the study as controls for healthy term pregnancy are given in Supplementary Data S1.

### Placental sampling

First trimester placental samples were obtained immediately after elective (surgical) termination of pregnancy (ETP) or uterine curettage due to incomplete or missed pregnancy loss (RPL samples). For two RPL samples and all ETP samples the maternal cells were removed under a stereomicroscope (Discovery V8, Zeiss) and the purified chorionic villous samples were karyotyped using conventional cytogenetic analysis to confirm normal male or female karyotype (United Laboratories, Tartu University Hospital). Part of the harvested chorionic villi containing both cyto- and syncytiotrophoblast cells of fetal origin were placed into a dry tube and stored at −80 °C until DNA extraction during a few days. The rest of the RPL placental samples (n = 8) were handled using the following protocol: washing with Dulbecco’s Phosphate Buffered Saline (PBS) solution to remove the maternal blood, immediate placing into a dry tube and storage at −80 °C without any further manipulation. For these samples, the presence of gross chromosomal aberrations was assessed using whole-genome CNV-profiling (Illumina GenomeStudio; Illumina Inc.; San Diego, CA). For recruited RPL and ETP cases, placental samples with aneuploidies were excluded from the current study.

For term placenta sampling, the full-thickness block of 2 cm was taken from a middle region of placenta (kept at + 4 °C) within 1 h after caesarean section or vaginal delivery. Collected tissue samples were washed with 1x PBS to remove contamination of maternal blood and placed immediately into a dry cryovial and stored at −80 °C for subsequent DNA extraction. All samples were collected by the same medical personnel. In all samples histological examination was carried out to confirm the non-malignancy of the tissues.

### Genome-wide SNP genotyping and CNV detection

The applied pipeline for CNV calling based on SNP-arrays has been originally described in the previous report^9^. Placental and blood genomic DNA was genotyped using Illumina HumanOmniExpress-24-v1 BeadChips (>713,000 markers with mean spacing 4.0 kb) at the institutional genotyping core facility (Estonian Genome Center; http://www.geenivaramu.ee/en). Samples were genotyped with an average overall call rate of 99.8% (median 99.8%). For each sample, calling of CNVs from genome-wide genotyping data was performed in parallel with three algorithms: QuantiSNP 2.3^42^, GADA (Genome Alteration Detection Analysis)^43^ and CNstream^44^. CNVs with QuantiSNP log Bayes Factor value < 5 were excluded from the resulting list of CNVs. In this project we analysed only autosomal CNVs. HD-CNV^45^ (Hotspot Detector for Copy Number Variants) was used to merge CNV regions. A criterion of 40% reciprocal overlap between parallel CNV calls was used to define two calls as identifying the same event. All CNVs called by at least two algorithms for the same individual in the same genomic loci were considered in the subsequent global analysis. CNV coordinates are according to human genome build 38 (hg38). An overall limited sample size and missing paternal data for seven RPL families did not allow reliable stratification of detected CNVs in the full RPL sample set into inherited and *de novo* somatic rearrangements. In detailed analysis of a subset of individual CNVs in RPL family trios the detected variants were assigned as inherited or somatic.

### Statistical analysis of CNVs

All statistical analyses were performed using R Statistical Software version 2.15.2 (http://www.r-project.org/). Data was tested for normality with Shapiro-Wilk normality test. *P*-values were estimated by Welch Two Sample t-test or non-parametric Wilcoxon rank sum test. A CNVR was defined when at least two CNVs representing the same type of genomic rearrangement (deletion or duplication) overlapped with 40% coverage of at least one of the involved CNVs. Subtelomeric regions were defined as proximal segments at both ends of the chromosome covering 5 percent of the overall chromosome length. Pericentromeric regions were defined to extend to both sides of the centre of the centromere up to 5 percent of the chromosomal length. Fisher’s exact test was used to assess differences among the study groups for the number of shared/unique CNVRs and large pericentromeric/subtelomeric CNVs. Results with *P*-values < 0.05 were considered significant.

### Functional enrichment analysis

Analysis was carried out for placental CNVRs using g:Profiler gGOSt web-based software (http://biit.cs.ut.ee/gprofiler/)^46^. Enrichment was tested for the functional categories defined in Gene Ontology (GO) and for the transcription binding site motifs derived from the TRANSFAC database. The analysis used a conservative output function ‘Best per parent group (strong)’. The analysis criteria to claim statistical significance applied g:SCS threshold as recommended. In the current study, we considered statistically significant enrichment, when adjusted *P* < 0.01.

### RNA-Seq dataset and analysis of the effect of placental CNVs on gene expression

The analysed 50 RNA-Seq datasets represent transcriptomes of 10 chorionic villous samples from normal 1^st^ trimester electively terminated pregnancies (ETP, n = 8) and two recurrent pregnancy loss (2^nd^ index cases of RPL71, RPL89; Supplementary Dataset 1), as well as 40 term gestation placentas. The RNA-Seq datasets have been generated for the same placental samples that were analysed for their CNV profile in the current study and in the previous report^9^. The detailed description of these RNA-Seq datasets is provided in recent publications from our group focusing on human placental transcriptome in normal and complicated pregnancies^20^,^21^. Condensed overview on the technical details of the applied RNA-Seq approach is provided in Supplementary Methods. Analysis for the effect of CNVs on the expression of the involved genes was conducted in parallel for all placental samples with available RNA-Seq data^20^,^21^ (n = 50) and separately in term pregnancy samples^20^ (n = 40) only. The analysis was restricted to the genes having concordant id in Ensembl v67 and Ensembl v87 (n = 47,429). Start and end coordinates (hg38) of CNV regions (n = 1,257 across all samples; n = 1094 in term samples) were compared to gene coordinates from Ensembl v87. Genes with any overlap with CNV regions were classified as ‘CNV genes’ (n = 2,273 in all samples; n = 2,163 in term samples). ‘CNV genes’ were subdivided as genes present only in deletion CNVs (n = 1,148 in all samples; n = 1,112 in term samples), present only in duplication CNVs (n = 1,063 in all samples; n = 1,010 in term samples). Genes detected in both deletion and duplication CNVs (n = 62 all samples; n = 41 term samples) were not analysed separately due to their small number. Median FPKM (fragments per kilobase per million) values in all samples and term samples were extracted for each gene. Differences in median FPKM distributions between gene groups were tested using Wilcoxon rank sum tests and compared visually on density plots generated with Gaussian kernel (smoothing bandwidth was selected using biased cross-validation as implemented in R).

## Additional Information

**How to cite this article:** Kasak, L. *et al*. Copy number variation profile in the placental and parental genomes of recurrent pregnancy loss families. *Sci. Rep.* **7**, 45327; doi: 10.1038/srep45327 (2017).

**Publisher's note:** Springer Nature remains neutral with regard to jurisdictional claims in published maps and institutional affiliations.

## Supplementary Material

Supplementary Materials

Supplementary Dataset 1

## Figures and Tables

**Table 1:** Comparative profile of placental and parental autosomal CNVs.

		Parental genome		Placental genome			
		RPL cases	Controls	RPL Pregnancy loss	RPL Live birth at term	Normal 1^st^ trimester pregnancy	Normal Term pregnancy
No of samples		25	24	10	3	9	8
No of CNVs per sample	all	7.0 (3–28)	10.0 (2–15)	9.0 (5–22)^*ab*^	7.0 (5–12)^*b*^	15.0 (4–39)	31.0 (17–94)
	dupl	2.0 (0–17)	2.0 (0–6)	2.5 (1–7)^*ab*^	3.0 (1–5)^*ab*^	8.0 (2–26)	25.5 (7–52)
	del	6.0 (1–13)	7.0 (1–12)	6.0 (2–20)	4.0 (4–7)	8.0 (1–15)	12.5 (4–56)
Cumulative span of all CNVs per sample, Mb	all	0.7 (0.0–2.8)	0.6 (0.0–1.8)	0.6 (0.3–3.5)^*b*^	0.9 (0.3–1.0)^*ab*^	1.8 (0.2–4.5)	4.4 (0.8–16.8)
	dupl	0.4 (0.0–2.0)	0.2 (0.0–1.6)	0.3 (0.0–0.5)^*ab*^	0.3 (0.3–0.3)^*ab*^	1.7 (0.1–3.5)	3.5 (0.8–5.8)
	del	0.3 (0.0–1.4)	0.3 (0.0–0.8)	0.3 (0.1–3.2)	0.7 (0.0–0.7)	0.3 (0.3–1.7)	1.3 (0.1–11.0)

Data are given as median (range). Group ‘Controls’ for the parental genome analysis consists of couples with normal term pregnancies and women who had undergone elective termination of pregnancy (ETP). Parental and placental datasets for ETP and term pregnancy controls have been derived from^9^. *P*-values for the differences among the groups were calculated by Welch two-sample t-test/Wilcoxon rank sum test. Statistically significant result (*P* < 0.05) compared to ^*a*^1^st^ trimester; ^*b*^term placental samples. All, pooled duplication and deletion CNVs; dupl, duplication; del, deletion; RPL, recurrent pregnancy loss.

**Table 2:** Shared placental autosomal CNVRs with alternative rearrangements in RPL and normal pregnancy groups.

Chr:start-end (hg38)^a^	Size (kb)	Type	Inherited/somatic	Group	Genes (GENCODE v24)
1:38885070–39240202	355.1	del	somatic	Term	*RHBDL2, AKIRIN1, NDUFS5, MACF1*
1:38992187–39062399	70.2	del	pat or somatic	RPL	*AKIRIN1, NDUFS5*
1:103603935–103668424	64.5	del	mat	Term	*AMY2A, AMY1A*
1:103603935–103613322	9.4	del	mat	1^st^ trimester	intergenic
1:103611243–103613322	2.1	del	pat or somatic	RPL	intergenic
2:51684073–51701068	17	dupl	somatic	Term	*AC007682.1*
2:51699461–51699766	0.3	del	somatic	Term	*AC007682.1*
2:51699461–51699766	0.3	del	somatic	RPL	*AC007682.1*
2:51699461–51699766	0.3	del	pat or somatic	RPL	*AC007682.1*
2:52097022–52248889	151.9	dupl	pat or somatic	RPL	*AC007682.1*
2:52099453–52142599	43.1	dupl	pat or somatic	1^st^ trimester	*AC007682.1*
2:52110968–52142599	31.6	dupl	pat or somatic	1^st^ trimester	*AC007682.1*
5:7843098–7946149	103.1	dupl	pat or somatic	1^st^ trimester	*C5orf49, FASTKD3, MTRR*
5:7859320–7927047	67.7	dupl	somatic	Term	*FASTKD3, MTRR*
5:7876175–7936174	60	dupl	pat or somatic	RPL	*MTRR*
6: 55911786–56388992	477.2	del	pat	Term	*COL21A1*
6: 55963929–55981729	17.8	del	mat	RPL	intergenic
10:66312326–66354723	42.4	del	mat	1^st^ trimester	*CTNNA3*
10:66747663–66804808	57.1	del	mat	RPL	*CTNNA3*
10:67085474–67128733	43.3	dupl	somatic	Term	*CTNNA3, LRRTM3*
10:67085474–67138548	53.1	dupl	pat or somatic	1^st^ trimester	*CTNNA3, LRRTM3*
11:3708446–3795547	87.1	del	pat or somatic	RPL	*NUP98*
11:3708446–3860500	152.1	del	pat or somatic	RPL	*NUP98, PGAP2, RHOG, STIM1, mir4687*
11:3828480–4147112	318.6	del	somatic	Term	*RHOG, STIM1, mir4687, RRM1, RRM1-AS1*
11:24391074–24529563	138.5	dupl	pat or somatic	1^st^ trimester	*LUZP2*
11:24626779–24644439	17.7	del	pat or somatic	RPL	*LUZP2*

^a^Chromosomal coordinates are provided according to the human genome assembly hg38. For some identified CNVs, the genome coordinates do not overlap between the listed placental samples, but coincide with the same gene. Chr, Chromosome; mat, maternally inherited; pat, paternally inherited; pat or somatic, only maternal CNV profile was available; RPL, placental sample from a miscarriage of RPL cases; Term, placental sample from normal term pregnancy ending with a live birth.

**Table 3:** Distribution of autosomal CNVs in the parental genomes of RPL cases compared to controls with no history of recurrent pregnancy loss.

CNVs	RPL cases n = 25	CNVs per genome	Controls n = 24	CNVs per genome
All	220	8.80	218	9.08
>300 kb	19	0.76	9	0.38
Pericentromeric	8	0.32	3	0.13
Subtelomeric	4	0.16	0	0

Control group consists of couples, who had normal term pregnancies (n = 8) and women who had undergone elective termination of uncomplicated 1^st^ trimester pregnancy (n = 8).

**Table 4:** Characteristics of the study groups.

ID (RPL type)	Age at index pregnancy (yrs): F, M	Live births prior index pregnancy (n)	Miscarriages prior index pregnancy (n)	Available placental samples (n)	Index pregnancy: gest. age (d)
*Recurrent pregnancy loss female patients*^a^*: the index pregnancy ending with a miscarriage*					
RPL18 (prim)	34, 37	0	7	1	130
RPL63^b^ (sec)	42, 46	1	4	n.a.	84
RPL69 (sec)	25, 26	1	4	n.a.	41
RPL71^c^ (sec)	37/38, 33/34	1	3/5	2	53/44
RPL89^c^ (prim)	31/32, 30/31	0	5/6	2	44/67
RPL30 (sec)	32, n.a.	1	2	1	49
RPL70 (sec)	34, n.a.	2	2	1	119
*Recurrent pregnancy loss couples: the index pregnancy ending with a miscarriage*					
RPL7^b^ (prim)	30, 35	0	2	n.a.	54
RPL16 (prim)	31, 40	0	2	n.a.	47
RPL45^b^ (prim)	37, 32	0	2	n.a.	63
RPL50 (prim)	32, 31	0	2	1	56
RPL60 (sec)	31, 33	2	2	1	45
RPL67 (prim)	29, 30	0	3	1	42
*Recurrent pregnancy loss couples: the index pregnancy ending with a live birth*					
RPL3^b^ (sec)	36, 39	2	3	1	286
RPL11^b^ (prim)	23, 24	0	3	1	287
RPL12^b^ (prim)	34, 29	0	4	1	284
*Reference data for normal pregnancies (8 1*^*st*^ *trimester women, 8 term pregnancy couples*)					
1^st^ trimester pregnancy	27 (18–33),n.a.	1 (0–3)	0 (0–0)	9	60 (51–81)
Term pregnancy	33 (18–37), 34 (22–38)	1 (0–2)	0 (0–1)	8	284 (260–291)

^a^Paternal blood DNA samples were not available for genotyping;

^b^parental blood DNA samples were analysed in a previous CNV study using a low resolution SNP array^7^, but were re-genotyped in the current study (see Methods);

^c^available placental samples from two miscarriages were analysed. Reference data are given as median (range) and include maternal-placental duos for the 1^st^ trimester and maternal-paternal-placental trios for the term pregnancies. d, days; F, female partner; M, male partner; n, number; n.a., not available; prim, primary RPL (no live births); sec, secondary RPL (occurrence of consecutive miscarriages after a live birth); yrs, years.
