# Supplementary material for: Copy number variation profile in the placental and parental genomes of recurrent pregnancy loss families
Source: Sci Rep. 2017 Mar 27;7:45327. doi: 10.1038/srep45327 (PMC5366903; doi:10.1038/srep45327)
Supplement: Supplementary Materials [file srep45327-s1.pdf]

Supplementary Materials

Copy number variation profile in the placental and parental genomes of recurrent pregnancy loss families

Laura Kasak, MSc[1], Kristiina Rull, MD, PhD[1,2,3], Siim Sõber[1,4], Maris Laan, PhD[1,4*]

[1]Human Molecular Genetics Research Group, Institute of Molecular and Cell Biology, University of Tartu, Riia 23 St., Tartu 51010, Estonia

[2]Department of Obstetrics and Gynaecology, University of Tartu, Puusepa St. 8, Tartu 51014, Estonia

[3]Women's Clinic of Tartu University Hospital, Puusepa St. 8, Tartu 51014, Estonia

[4]Institute of Biomedicine and Translational Medicine, University of Tartu, Ravila St. 19, Tartu 51014, Estonia

[*]Corresponding author: maris.laan@ut.ee

SUPPLEMENTARY METHODS

Re-analysis of a subset of RPL patients with a SNP array with increased resolution

Four RPL couples and two RPL female patients included into the current study had been previously analysed in our seminal report assessing the role of CNVs in predisposing to RPL[1]. As this previous study was based on a lower-resolution SNP array (Illumina Human370CNV-Quad; 370 000 markers) compared to the current study design (Illumina HumanOmniExpress-24-v1 BeadChips; >713 000 markers), these samples were re-genotyped to enable uniform CNV calling of all samples analysed in the current study. Mean spacing between SNPs on the HumanOmniExpress-24-v1 BeadChips (4.0 kb) is nearly twofold shorter compared to Human370CNV-Quad array (7.7 kb). Across 10 analysed parental genomes, 38% of CNVs (16 duplications and 18 deletions) identified in the current study had been also detected by previous analysis using a SNP array with lower resolution. Denser spacing of SNP markers enabled identification of additional 18 duplications and 36 deletions for these 10 RPL patients. Whereas the median length of previously and additionally detected duplication CNVs differed nearly two times (155.5 (range: 45.6 – 655.5) *vs* 81.7 kb (range: 8.1 – 895.8)), the uncovered additional deletions were substantially shorter than those also detected by the low-resolution array (11.7 (range: 0.6 – 204.2) *vs* 72.8 kb (range: 0.9 – 563.2)). This confirms that the use of higher density SNP array increases the ability to detect smaller structural genomic variants.

RNA-Seq dataset

The analysed 50 RNA-Seq datasets represent transcriptomes of 10 chorionic villous samples from normal 1st trimester electively terminated pregnancies (ETP, n=8) and recurrent pregnancy loss (cases RPL71, RPL89; Supplmentary Dataset 1), as well as 40 term gestation placentas. The RNA-Seq datasets have been generated for the same placental samples that

were analysed for their CNV profile in the current study and in the previous report[2]. The detailed description of these RNA-Seq datasets is provided in recent publications from our group focusing on human placental transcriptome in normal and complicated pregnancies[3,4]. In brief, total RNA was extracted from 200–300 mg of homogenized placental tissue using TRIzol reagent (Invitrogen, Life Technologies). The median RIN value for 50 RNA samples was 7.05 (range 5.9-8.9). To produce RNA-Seq libraries total RNA was further purified with RNeasy MinElute columns (Qiagen, Netherlands) according to the manufacturers' protocol. High quality DNA-free total RNA (5 μ g) was used for rRNA depletion (Ribo-Zero™ rRNA Removal Kit, Epicentre) and library preparation with cDNA synthesis (Life Technologies) followed by Nextera™ Technology (Illumina). Initial RNA-Seq data analysis and preparation was conducted by the RNA-Seq pipeline v2.4 (Sequencing Unit, FIMM Technology Centre) consisting of FastQC version 0.10.0 for quality control; reads were filtered for adaptor, rRNA and mtDNA sequences as well as homopolymer stretches using custom python scripts. Reads were aligned to human genome assembly (GRCh37.p7/hg19) with TopHat version 2.0.3. Transcript quantification was conducted with Cufflinks v 2.0.2 with reference annotation (measured as FPKM) and gene expression was quantified by htseq-count (as raw read counts). Full clinical details as well as specifics of library preparation, RNA-Seq and basic bioinformatics of raw data are available in[3,4].

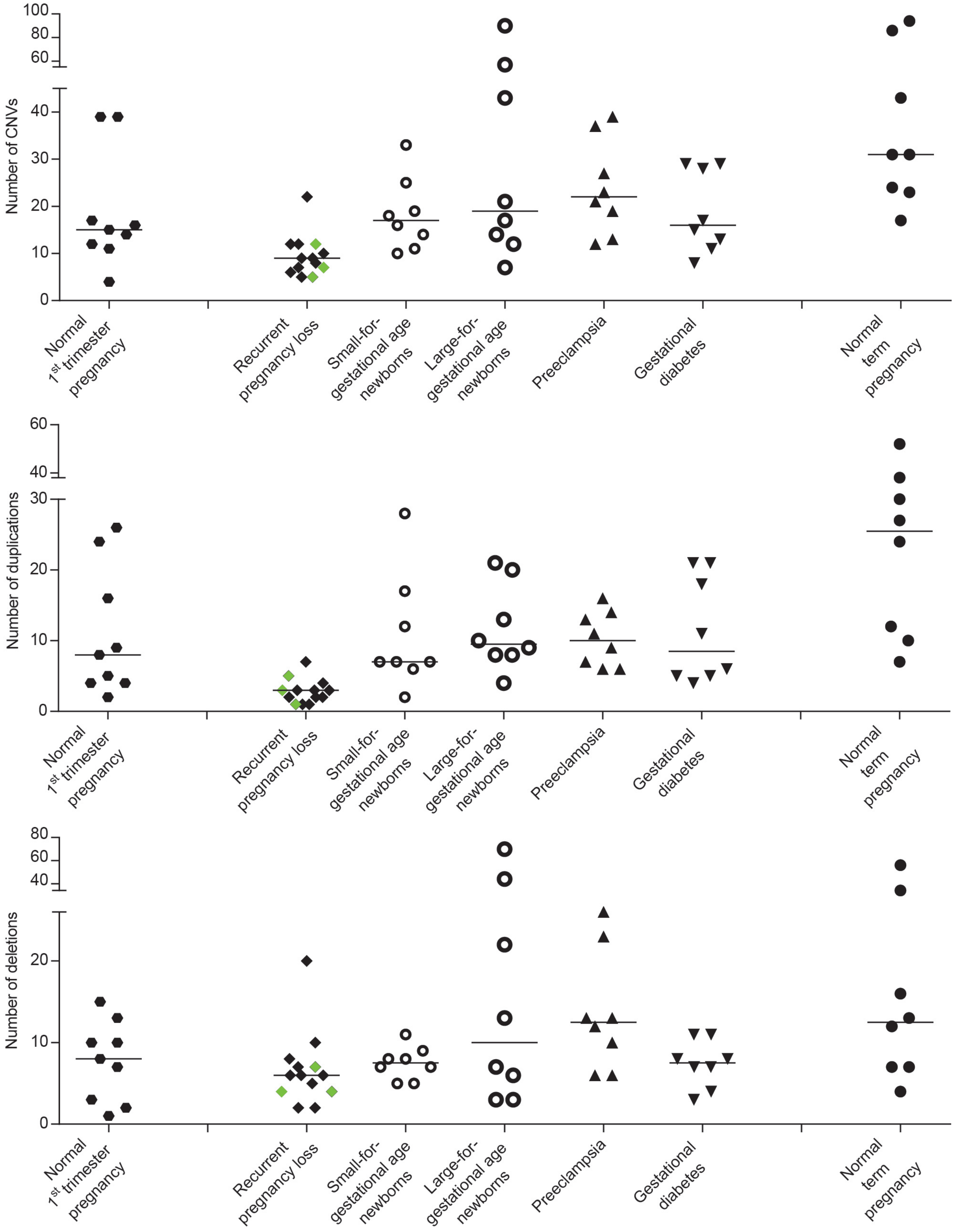

Supplementary Figure S1. Number of all autosomal CNVs, duplications and deletions in the placental samples of recurrent pregnancy loss (RPL) cases compared to controls representing normal 1st trimester, normal term pregnancy and late pregnancy complications (data from[2]). Analysed late pregnancy complications include preeclampsia, gestational diabetes and fetal growth disturbances (small- and large-for gestational age newborns). Placentas from RPL couples eventually succeeding in a term live birth after a successful pregnancy are indicated with green diamonds.

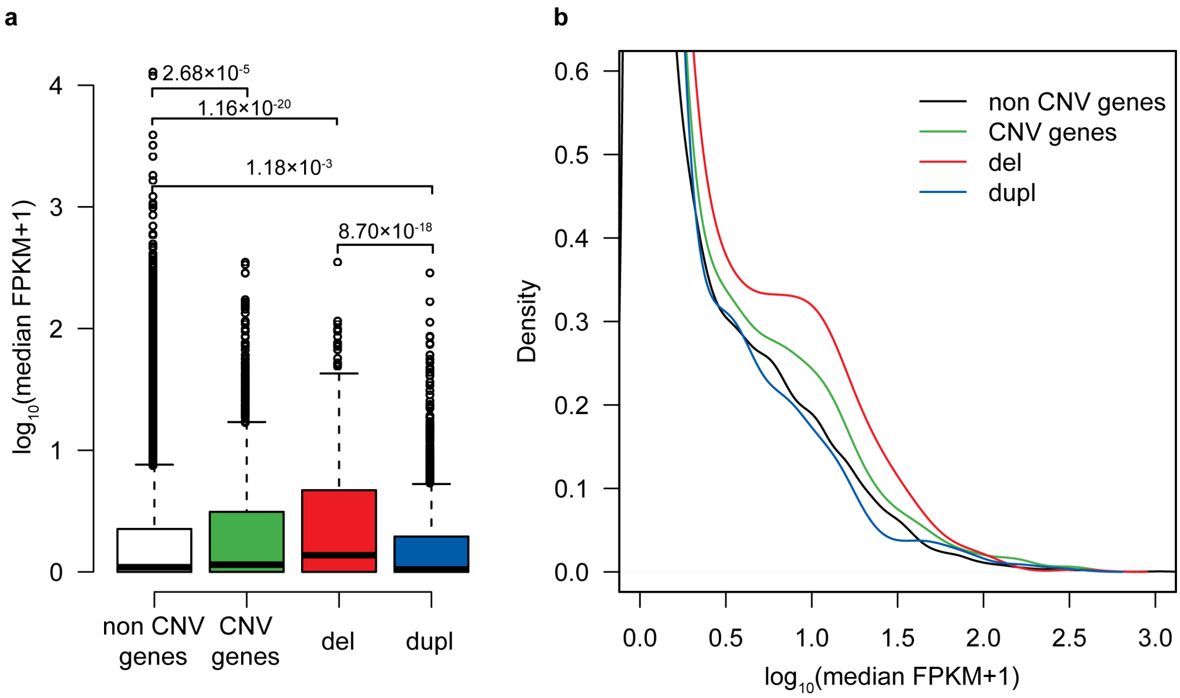

Supplementary Figure S2. **Profiles of median expression values of genes overlapping with CNVs (‘CNV genes’; n=2,163) compared to genes not overlapping with CNVs (‘non CNV genes’; n=45,266) in placental samples collected at term** (n=40; RNA-Seq dataset details in reference[3] and Supplementary Methods). Expression levels are measured as FPKM (fragments per kilobase per million) and distributions are shown as boxplots **(a)** and plotted density estimates with Gaussian kernel **(b)**. Genes affected only by deletion CNVs (del; n=1,112) and duplication CNVs (dupl; n=1,010) are shown separately. *P*-values for between-group comparisons are derived from Wilcoxon rank sum tests.

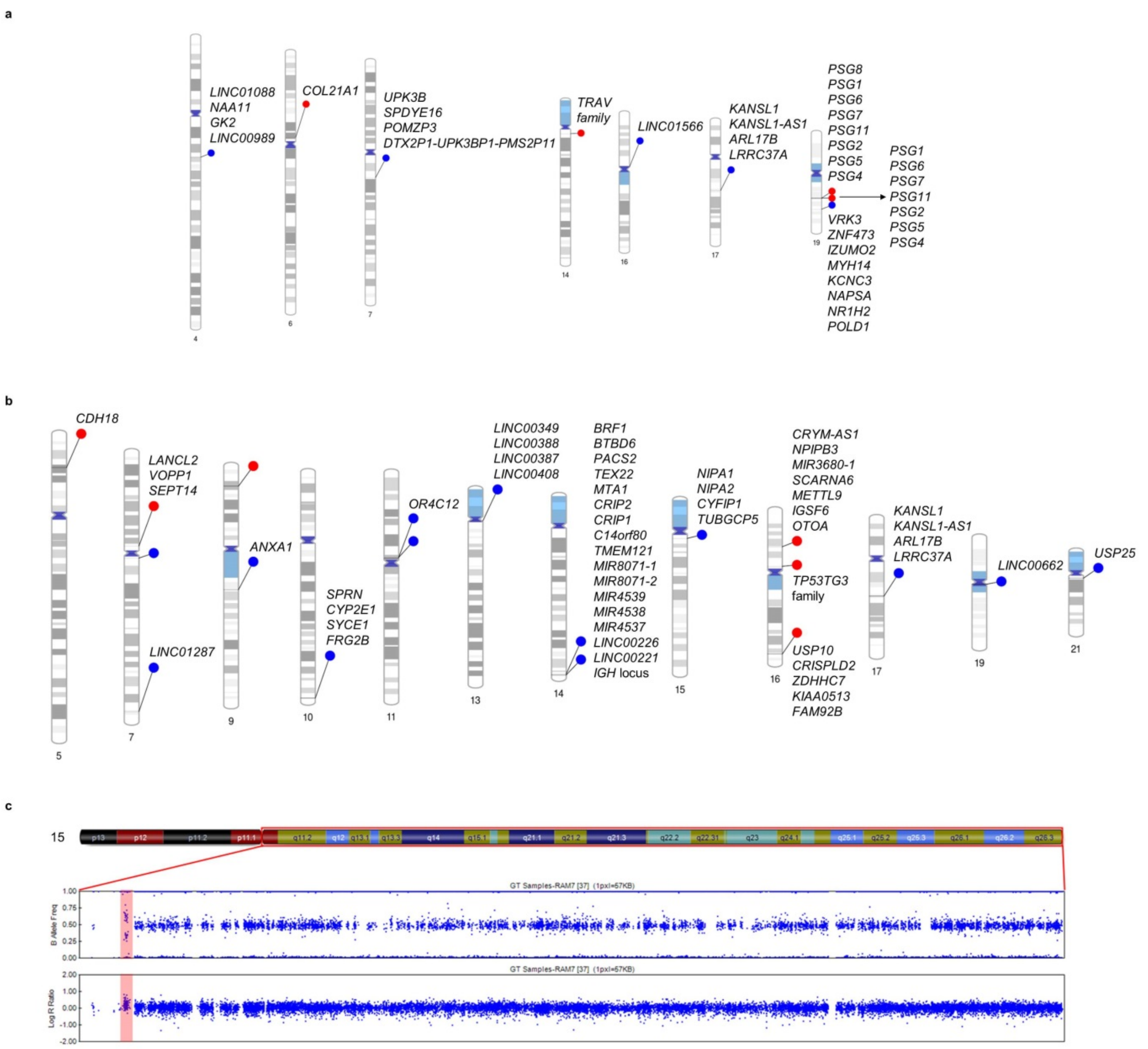

Supplementary Figure S3. Chromosomal distribution of large, >300 kb autosomal CNVs in the parental genomes of (a) controls and (b) RPL cases. Dots indicate locations of duplication (blue) and deletion (red) CNVs. Large CNVs in the RPL parental genomes were prevalently (63% of all >300 kb CNVs) pericentromeric or subtelomeric. Control group consists of cases who had normal term pregnancies (n=8) and women who had undergone elective termination of uncomplicated 1st trimester pregnancy (n=8). http://visualization.ritchielab.psu.edu/phenograms/plot. (**c**) B allele frequency and Log R ratio graphs for 15q of male partner of the couple RPL7 (Illumina GenomeStudio). Red boxes indicate the duplication CNV at 15q11.2.

Supplementary Table S1. Comparative profile of placental and parental autosomal CNVs. Data are given as mean ± SD. Group 'Controls' for the parental genome analysis consists of couples with normal term pregnancies and women who had undergone elective termination of pregnancy (ETP). Parental and placental datasets for ETP and term pregnancy controls have been derived from[2]. *P*-values for the differences among the groups were calculated by Welch two-sample t-test/Wilcoxon rank sum test. Statistically significant result ($P < 0.05$) compared to [a]1st trimester; [b]term placental samples; [c]RPL parental genomes. All, pooled duplication and deletion CNVs; dupl, duplication; del, deletion; RPL, recurrent pregnancy loss.

		Parental genome		Placental genome			
		RPL cases	Controls	RPL Pregnancy loss	RPL Live birth at term	Normal 1st trimester pregnancy	Normal Term pregnancy
No of samples		25	24	10	3	9	8
No of CNVs per sample	all	8.8 ± 5.4	9.2 ± 3.6	10.0 ± 4.8[ab]	8.0 ± 3.6[b]	18.6 ± 12.2	43.6 ± 29.7
	dupl	3.0 ± 3.5	2.5 ± 1.5	2.8 ± 1.8[ab]	3.0 ± 2.0[ab]	10.9 ± 9.0	25.0 ± 15.3
	del	5.8 ± 3.0	6.6 ± 2.7	7.2 ± 5.1	5.0 ± 1.7	7.7 ± 4.9	18.6 ± 17.7
Cumulative span of all CNVs per sample, Mb	all	0.9 ± 0.6	0.7 ± 0.5	1.1 ± 1.0[b]	0.8 ± 0.4[ab]	2.2 ± 1.5	5.7 ± 5.2
	dupl	0.5 ± 0.5	0.4 ± 0.4	0.3 ± 0.2[ab]	0.3 ± 0.0[ab]	1.6 ± 1.1	3.0 ± 2.0
	del	0.4 ± 0.3	0.3 ± 0.2	0.7 ± 1.0	0.5 ± 0.4	0.6 ± 0.6	2.7 ± 3.7
CNV length across samples, kb	all	97.0 ± 170.2	72.6 ± 131.5	105.5 ± 150.2[b]	94.6 ± 147.1[b]	119.4 ± 193.1	130.5 ± 169.8
	dupl	160.2 ± 218.3	144.0 ± 200.9	109.9 ± 106.9	91.7 ± 100.1	150.4 ± 229.4	120.7 ± 184.5
	del	63.7 ± 127.0	45.6 ± 78.1	103.8 ± 164.6[bc]	96.3 ± 172.7[b]	75.5 ± 112.3	143.5 ± 147.4

Supplementary Table S2. Copy number variants identified based on Illumina HumanOmniExpress array genotyping and CNV calling in placental (n=13), maternal and paternal blood DNA (n=25). The analysis included only autosomal CNVs. Genomic coordinates are given according to hg38. Chr, chromosome; Dupl, duplication; Del, deletion.

Chr	Start (hg38)	End	Length	Group	Type
1	1334343	1359943	25600	mother	Dupl
1	2427505	2428542	1037	RPL pregnancy loss placenta	Dupl
1	2427505	2434877	7372	RPL pregnancy loss placenta	Dupl
1	3451579	3734754	283175	RPL live birth placenta	Dupl
1	9261182	9340809	79627	mother	Dupl
1	10606460	10680569	74109	RPL pregnancy loss placenta	Dupl
1	10612878	10679198	66320	RPL live birth placenta	Dupl
1	38992187	39062399	70212	RPL pregnancy loss placenta	Del
1	70542243	70553303	11060	RPL pregnancy loss placenta	Del
1	70542243	70553303	11060	father	Del
1	70542243	70553303	11060	father	Del
1	70542243	70553303	11060	mother	Del
1	84321629	84370016	48387	RPL pregnancy loss placenta	Del
1	103611243	103613322	2079	RPL live birth placenta	Del
1	103611243	103613322	2079	RPL pregnancy loss placenta	Del
1	103611243	103613322	2079	father	Del
1	103611243	103613322	2079	mother	Del
1	103611243	103613322	2079	mother	Del
1	162762701	162769219	6518	mother	Del
1	205029247	205038822	9575	father	Del
1	212465661	212487339	21678	RPL pregnancy loss placenta	Del
1	220053390	220126693	73303	RPL pregnancy loss placenta	Del
1	224112847	224343857	231010	RPL pregnancy loss placenta	Del
1	231573658	231677388	103730	father	Dupl
1	238929939	238932730	2791	RPL live birth placenta	Del
1	248357730	248744377	386647	RPL pregnancy loss placenta	Dupl
2	1979030	1985517	6487	mother	Del
2	1979030	1985517	6487	mother	Del
2	38727987	38750470	22483	RPL pregnancy loss placenta	Dupl
2	51699461	51699766	305	RPL pregnancy loss placenta	Del
2	51699461	51699766	305	RPL pregnancy loss placenta	Del
2	51699461	51699766	305	father	Del
2	52097022	52248889	151867	RPL pregnancy loss placenta	Dupl
2	57172695	57218336	45641	father	Dupl
2	57172695	57256534	83839	father	Dupl
2	67405082	67413146	8064	mother	Dupl
2	76701093	76721975	20882	father	Del
2	76713923	76721975	8052	mother	Del
2	99439044	99445701	6657	father	Dupl

Chr	Start (hg38)	End	Length	Group	Type
2	109720215	110222769	502554	RPL live birth placenta	Del
2	154008968	154029732	20764	mother	Del
2	168499504	168505032	5528	RPL pregnancy loss placenta	Del
2	183933099	183937882	4783	RPL live birth placenta	Del
2	212318971	212327544	8573	father	Del
2	212322309	212325270	2961	father	Del
2	212322309	212327544	5235	mother	Del
2	218378139	218385280	7141	RPL pregnancy loss placenta	Del
2	233818079	233829877	11798	mother	Dupl
2	238114887	238167171	52284	mother	Dupl
2	238114887	238167171	52284	mother	Dupl
2	238116989	238167171	50182	father	Dupl
2	238116989	238170901	53912	mother	Dupl
2	238139269	238167171	27902	RPL pregnancy loss placenta	Dupl
2	238139269	238167294	28025	father	Dupl
2	241931649	242106609	174960	father	Del
2	241931649	242106609	174960	father	Del
2	241975583	242106609	131026	RPL pregnancy loss placenta	Del
2	241975583	242106609	131026	RPL pregnancy loss placenta	Del
2	241975583	242106609	131026	mother	Del
2	241975583	242106609	131026	mother	Del
2	241975583	242106609	131026	mother	Del
3	19817	102299	82482	father	Dupl
3	19817	90294	70477	mother	Dupl
3	1515729	1680597	164868	mother	Dupl
3	1741650	1745897	4247	RPL pregnancy loss placenta	Del
3	5349869	5396688	46819	RPL pregnancy loss placenta	Del
3	5349869	5396688	46819	mother	Del
3	5358483	5396688	38205	mother	Del
3	9321588	9325935	4347	father	Del
3	58999352	59073179	73827	mother	Del
3	62972894	63007407	34513	mother	Del
3	62981195	63002132	20937	mother	Del
3	65206172	65228858	22686	RPL pregnancy loss placenta	Del
3	65206172	65228858	22686	father	Del
3	65206172	65228858	22686	mother	Del
3	75339010	75587298	248288	mother	Del
3	75379524	75587298	207774	father	Del
3	113857355	113901025	43670	mother	Del
3	130044038	130058958	14920	RPL pregnancy loss placenta	Del
3	130044038	130058958	14920	mother	Del
3	138088417	138099257	10840	RPL pregnancy loss placenta	Dupl
3	143447087	143476613	29526	father	Del

Chr	Start (hg38)	End	Length	Group	Type
3	151770545	151828253	57708	mother	Del
3	151796802	151828253	31451	RPL pregnancy loss placenta	Del
3	151796802	151828253	31451	RPL pregnancy loss placenta	Del
3	151796802	151828253	31451	father	Del
3	151796802	151856498	59696	mother	Del
3	155752796	155800254	47458	father	Dupl
3	159558237	159580247	22010	mother	Del
3	162413620	162424687	11067	mother	Del
3	165323328	165350331	27003	mother	Del
3	176175294	176189554	14260	father	Del
3	186668987	186715257	46270	RPL pregnancy loss placenta	Del
3	186668987	186693287	24300	mother	Del
4	1010571	1051477	40906	mother	Dupl
4	2243138	2259993	16855	RPL pregnancy loss placenta	Dupl
4	2248382	2259993	11611	RPL live birth placenta	Dupl
4	3720680	3873507	152827	RPL live birth placenta	Dupl
4	9369390	9649789	280399	father	Dupl
4	11545482	11640910	95428	mother	Del
4	32016212	32223858	207646	mother	Dupl
4	35148038	35149615	1577	RPL pregnancy loss placenta	Del
4	35148038	35149615	1577	father	Del
4	52822821	52844661	21840	mother	Del
4	63272623	63284948	12325	mother	Del
4	63272623	63284948	12325	mother	Del
4	93518875	93713151	194276	father	Del
4	121364108	121367490	3382	father	Del
4	160147355	160149907	2552	father	Del
4	164655588	164665848	10260	RPL live birth placenta	Del
4	164655588	164665848	10260	mother	Del
4	171616240	171630345	14105	RPL pregnancy loss placenta	Del
5	788531	840602	52071	mother	Del
5	2041551	2064855	23304	mother	Del
5	7178531	7189812	11281	father	Del
5	7178531	7190961	12430	mother	Del
5	7876175	7936174	59999	RPL pregnancy loss placenta	Dupl
5	8707907	8744445	36538	RPL live birth placenta	Del
5	8707907	8743278	35371	mother	Del
5	8707907	8744445	36538	mother	Del
5	18798936	18917719	118783	father	Dupl
5	19932577	20248273	315696	father	Del
5	20859532	20878154	18622	mother	Del
5	97712762	97781411	68649	RPL live birth placenta	Del
5	97712762	97775819	63057	RPL pregnancy loss placenta	Del

Chr	Start (hg38)	End	Length	Group	Type
5	97712762	97763616	50854	father	Del
5	97712762	97775819	63057	mother	Del
5	100051362	100055023	3661	mother	Del
5	106893823	106895197	1374	mother	Del
5	106893823	106895197	1374	mother	Del
5	131681245	131740228	58983	RPL live birth placenta	Del
5	131681245	131922685	241440	mother	Del
5	139585791	139613118	27327	RPL pregnancy loss placenta	Del
5	177812493	177949316	136823	mother	Del
5	179292363	179509721	217358	father	Dupl
6	4012695	4016114	3419	RPL pregnancy loss placenta	Del
6	25320692	25330310	9618	mother	Del
6	31253262	31254137	875	father	Del
6	55963929	55981729	17800	RPL pregnancy loss placenta	Del
6	55963929	55981729	17800	mother	Del
6	55963929	55981729	17800	mother	Del
6	66307601	66337401	29800	father	Del
6	66307601	66337401	29800	father	Del
6	74806987	74811977	4990	RPL pregnancy loss placenta	Del
6	76606071	76617085	11014	mother	Del
6	79848763	79862766	14003	father	Del
6	94228015	94355946	127931	RPL pregnancy loss placenta	Del
6	94228015	94362201	134186	mother	Del
6	98347456	98479487	132031	RPL pregnancy loss placenta	Del
6	98371924	98466804	94880	mother	Del
6	131407118	131577068	169950	mother	Dupl
6	137578655	137644281	65626	RPL live birth placenta	Dupl
7	1543652	1550807	7155	father	Dupl
7	17360234	17418454	58220	RPL pregnancy loss placenta	Del
7	17386271	17418454	32183	mother	Del
7	55274956	55838203	563247	father	Del
7	62583246	63238736	655490	father	Dupl
7	62583246	62652633	69387	father	Dupl
7	105197603	105436530	238927	RPL pregnancy loss placenta	Del
7	105307629	105637346	329717	RPL pregnancy loss placenta	Del
7	111252170	111385477	133307	RPL live birth placenta	Del
7	111252170	111383266	131096	father	Del
7	111337288	111683743	346455	RPL pregnancy loss placenta	Del
7	111433654	111540488	106834	father	Del
7	122959245	122995119	35874	mother	Del
7	132051593	132085456	33863	mother	Del
7	153245595	153834812	589217	mother	Dupl
8	5741877	5747565	5688	RPL pregnancy loss placenta	Del

Chr	Start (hg38)	End	Length	Group	Type
8	5741877	5747565	5688	father	Del
8	5741877	5747565	5688	mother	Del
8	5743669	5747565	3896	mother	Del
8	16090726	16172175	81449	RPL pregnancy loss placenta	Del
8	16090726	16163959	73233	RPL pregnancy loss placenta	Del
8	16092262	16168455	76193	mother	Del
8	76676313	76713074	36761	mother	Del
8	83020549	83056421	35872	mother	Dupl
8	91116612	91171430	54818	RPL pregnancy loss placenta	Del
8	91116612	91171430	54818	mother	Del
8	103411258	103416302	5044	mother	Del
8	103411258	103416302	5044	mother	Del
8	103411258	103416302	5044	mother	Del
9	318153	503735	185582	RPL pregnancy loss placenta	Dupl
9	318153	517715	199562	RPL pregnancy loss placenta	Dupl
9	569992	594579	24587	RPL pregnancy loss placenta	Del
9	7887951	7891408	3457	mother	Del
9	11825229	12227068	401839	father	Del
9	72953887	73364757	410870	mother	Dupl
9	93259834	93289897	30063	father	Dupl
9	126886252	126897639	11387	RPL live birth placenta	Del
9	133204076	133210396	6320	mother	Del
9	134393077	134411093	18016	RPL live birth placenta	Dupl
9	136561760	136624513	62753	father	Dupl
9	136592640	136624513	31873	RPL pregnancy loss placenta	Dupl
9	136592640	136624513	31873	RPL pregnancy loss placenta	Dupl
9	136592640	136624513	31873	mother	Dupl
9	136592640	136762254	169614	mother	Dupl
9	136592640	136624513	31873	mother	Dupl
9	136606165	136624513	18348	RPL live birth placenta	Dupl
9	136606165	136624513	18348	mother	Dupl
10	21390598	21469938	79340	RPL pregnancy loss placenta	Del
10	46172086	46332633	160547	RPL pregnancy loss placenta	Dupl
10	54161545	54229219	67674	mother	Del
10	66747663	66804808	57145	RPL pregnancy loss placenta	Del
10	66747663	66804808	57145	mother	Del
10	89184459	89185999	1540	RPL live birth placenta	Del
10	94739953	94797579	57626	mother	Del
10	105185478	105208911	23433	mother	Del
10	118826403	119033191	206788	RPL live birth placenta	Dupl
10	124609101	124723355	114254	RPL pregnancy loss placenta	Dupl
10	133421076	133563944	142868	father	Dupl
10	133421076	133740558	319482	mother	Dupl

Chr	Start (hg38)	End	Length	Group	Type
11	892760	900809	8049	mother	Dupl
11	2012869	2036417	23548	mother	Dupl
11	2365406	2426625	61219	RPL pregnancy loss placenta	Dupl
11	3708446	3795547	87101	RPL pregnancy loss placenta	Del
11	3708446	3860500	152054	RPL pregnancy loss placenta	Del
11	9163530	9518776	355246	RPL pregnancy loss placenta	Del
11	24626779	24644439	17660	RPL pregnancy loss placenta	Del
11	46188708	46216180	27472	RPL pregnancy loss placenta	Del
11	49955784	50709229	753445	mother	Dupl
11	50098683	50709229	610546	mother	Dupl
11	60095631	60100108	4477	RPL pregnancy loss placenta	Del
11	81792950	81802672	9722	RPL pregnancy loss placenta	Del
11	81794421	81802672	8251	RPL pregnancy loss placenta	Del
11	128653624	128666428	12804	RPL pregnancy loss placenta	Del
12	6134991	6138398	3407	mother	Del
12	7848316	7973149	124833	father	Dupl
12	8876276	8896637	20361	RPL pregnancy loss placenta	Del
12	19316386	19425173	108787	mother	Dupl
12	31031418	31253973	222555	RPL pregnancy loss placenta	Dupl
12	31088411	31256645	168234	RPL pregnancy loss placenta	Dupl
12	31088411	31256844	168433	RPL pregnancy loss placenta	Dupl
12	31088411	31253973	165562	mother	Dupl
12	31088411	31262057	173646	mother	Dupl
12	31113353	31255762	142409	mother	Dupl
12	31848581	31919612	71031	father	Dupl
12	49137675	49251457	113782	RPL pregnancy loss placenta	Del
12	61703812	61714867	11055	father	Dupl
12	63548869	63724778	175909	RPL pregnancy loss placenta	Dupl
12	82198117	82218638	20521	mother	Del
12	99608908	99614995	6087	father	Del
12	109111774	109225776	114002	mother	Dupl
12	128745968	128748468	2500	father	Del
12	128745968	128748468	2500	mother	Del
13	18484577	18943280	458703	mother	Dupl
13	36737194	36777619	40425	RPL pregnancy loss placenta	Del
13	36738009	36777619	39610	mother	Del
13	45605092	45641212	36120	mother	Del
13	68672890	68693849	20959	RPL pregnancy loss placenta	Del
13	68672890	68693849	20959	mother	Del
13	95284895	95380685	95790	father	Dupl
13	100014379	100134403	120024	RPL pregnancy loss placenta	Del
14	22270094	22471422	201328	father	Del
14	22270094	22473900	203806	mother	Del

Chr	Start (hg38)	End	Length	Group	Type
14	22270094	22473159	203065	mother	Del
14	23960040	24014851	54811	mother	Del
14	23984157	24014851	30694	RPL pregnancy loss placenta	Del
14	23984157	24014851	30694	mother	Del
14	44719763	44740193	20430	RPL pregnancy loss placenta	Del
14	44719763	44740193	20430	mother	Del
14	49623728	49678963	55235	father	Dupl
14	84582471	84606219	23748	father	Del
14	85821003	85843820	22817	father	Del
14	85821003	85843820	22817	mother	Del
14	87933144	87980532	47388	mother	Del
14	103595309	103642406	47097	RPL pregnancy loss placenta	Del
14	105156924	105207355	50431	mother	Dupl
14	105207888	106576619	1368731	mother	Dupl
14	105581568	105884536	302968	mother	Dupl
15	22584820	23122762	537942	father	Dupl
15	24152472	24170857	18385	mother	Del
15	25170910	25240618	69708	mother	Dupl
15	43544751	43647444	102693	mother	Del
15	45066648	45102208	35560	mother	Del
15	51841717	52057158	215441	RPL pregnancy loss placenta	Del
15	57352258	57366684	14426	RPL live birth placenta	Del
15	85968459	85977902	9443	mother	Del
16	370907	377479	6572	mother	Dupl
16	774098	795819	21721	mother	Dupl
16	1752231	1792208	39977	RPL pregnancy loss placenta	Dupl
16	1752231	1792208	39977	mother	Dupl
16	1752231	1792208	39977	mother	Dupl
16	21305426	21828019	522593	RPL live birth placenta	Del
16	21305426	21828019	522593	father	Del
16	32899200	33993220	1094020	RPL pregnancy loss placenta	Del
16	32899200	33993220	1094020	mother	Del
16	35363172	35512674	149502	RPL pregnancy loss placenta	Dupl
16	35372988	35512674	139686	RPL pregnancy loss placenta	Dupl
16	57016436	57026301	9865	mother	Del
16	76886122	76981412	95290	mother	Del
16	77959610	77963812	4202	RPL pregnancy loss placenta	Del
16	79230038	79239785	9747	mother	Del
16	80857994	80910771	52777	RPL live birth placenta	Del
16	81190041	81267966	77925	mother	Del
16	84775294	85133284	357990	father	Del
16	85975035	85981113	6078	mother	Del
16	88456148	88528983	72835	mother	Dupl

Chr	Start (hg38)	End	Length	Group	Type
16	90007593	90027787	20194	mother	Dupl
17	29243604	29482086	238482	RPL pregnancy loss placenta	Del
17	37318076	37488149	170073	RPL pregnancy loss placenta	Del
17	46092442	46272724	180282	father	Dupl
17	46092442	46272724	180282	father	Dupl
17	46092442	46272724	180282	mother	Dupl
17	46092442	46272724	180282	mother	Dupl
17	46092442	46502770	410328	mother	Dupl
17	46099737	46502770	403033	RPL pregnancy loss placenta	Dupl
17	75610194	75620784	10590	RPL pregnancy loss placenta	Dupl
17	77264304	77275705	11401	RPL pregnancy loss placenta	Del
18	10918245	10919991	1746	father	Del
18	44185713	44194278	8565	RPL pregnancy loss placenta	Del
18	60038756	60040758	2002	father	Del
18	69072212	69088271	16059	mother	Del
18	69077071	69088271	11200	father	Del
18	69080331	69088271	7940	father	Del
18	69080331	69088271	7940	mother	Del
18	69541905	69550035	8130	father	Del
19	730030	757412	27382	RPL pregnancy loss placenta	Dupl
19	730030	757412	27382	mother	Dupl
19	8995829	9191318	195489	RPL pregnancy loss placenta	Del
19	15653506	15654151	645	mother	Del
19	27428816	27752973	324157	father	Dupl
19	37034969	37043447	8478	mother	Del
19	40850846	40873243	22397	RPL live birth placenta	Del
19	41114514	41210366	95852	RPL pregnancy loss placenta	Del
19	43055178	43553616	498438	RPL pregnancy loss placenta	Del
19	43055178	43331618	276440	mother	Del
19	46294573	46313531	18958	father	Del
19	50757732	50760253	2521	RPL live birth placenta	Dupl
19	53429042	53508130	79088	mother	Del
19	54402316	54409551	7235	RPL pregnancy loss placenta	Del
20	388261	417284	29023	father	Dupl
20	4905844	5060783	154939	RPL pregnancy loss placenta	Del
20	12821422	12908666	87244	father	Dupl
20	15776678	15783700	7022	mother	Del
20	45252989	45272543	19554	mother	Del
20	48890385	49417294	526909	RPL pregnancy loss placenta	Del
20	63236102	63247657	11555	mother	Dupl
20	63532951	63570431	37480	mother	Dupl
21	15584547	15937614	353067	mother	Dupl
21	19934016	19963774	29758	father	Del

Chr	Start (hg38)	End	Length	Group	Type
21	30720114	30866332	146218	mother	Dupl
21	45504871	45533378	28507	RPL pregnancy loss placenta	Dupl
22	25272763	25518626	245863	RPL pregnancy loss placenta	Del
22	25275848	25518626	242778	RPL pregnancy loss placenta	Del

Supplementary Table S3. Gene enrichment analysis of placental CNVRs. Gene enrichment analysis implemented in g:Profiler software[5] and a conservative output function 'Best per parent group (strong)' was applied. Number of CNVRs entering the test was 339 for the control and 68 for the RPL group-specific analysis. Overrepresented functional categories in GO and TRANSFAC databases with highly significant statistical support are shown ($P<1 \times 10^{-2}$). RPL, recurrent pregnancy loss; GO, gene ontology; TF, transcription factor binding motif.

Type	ID	Name	No of genes in category	No of genes in query	No of common genes	Pathway genes (%)	Query genes (%)	Corrected *P*-value	Common genes
CNVRs only in the placental genomes of control 1st trimester and term pregnancies									
GO	0019814	immunoglobulin complex	14	595	7	50	1.2	2.00E-04	*IGHE, IGHG4, IGHG2, IGHA1, IGHG1, IGHD, IGHV3-23*
	0006958	complement activation, classical pathway	44	595	10	22.7	1.7	1.94E-03	*C7, C2, IGHE, IGHG4, IGHG2, IGHA1, IGHG1, IGHV3-23, C4B, C4A*
	0034987	immunoglobulin receptor binding	14	595	6	42.9	1	5.56E-03	*IGHE, IGHG4, IGHG2, IGHA1, IGHG1, IGHV3-23*
	0006910	phagocytosis, recognition	22	595	7	31.8	1.2	8.00E-03	*CD36, IGHE, IGHG4, IGHG2, IGHA1, IGHG1, IGHV3-23*
TF	M00716_1	ZF5; motif: GSGCGCGR	16480	630	510	3.1	81	3.27E-07	*RAD52, MAD1L1, HOXA11, THSD7A, RPUSD1, RHBDF1, PIGQ, HEATR5B, FYN, ZC3H3, TTC27, PLEKHB1, SPAST, KPNA6, LRRC7, HOXC8, JADE2, CNTLN, EPHA3, KCNQ1, ARID4B, SYNE2, FAM168A, MTA3, CCAR1, VMP1, DGKA, SYT1, INPP5A, MAST4, FGF10, CLTCL1, LIMS2, KCNQ2, SEMA3C, HOXA9, TP73, SMARCA2, ERC1, TRPM3, SLCO1A2, P3H2, RAB11FIP3, AARS, ZC3HC1, TRPM7, SNAP23, MFSD11, MSH2, PRODH, CRYBB2P1, HSCB, XBP1, PPP6R2, POLE2, CHGA, ITPK1, RAE1, PLCB4, RNF125, RIOK3, FGF14, MSLN, PHKB, NPRL3, MPG, NARFL, HAGHL,*

FAM173A, METRN, STUB1, RHBDL1, CAPN15, ZNF106, MYEF2, SGK3, TRPS1, GSDMD, PYCRL, EEF1D, TUSC3, HOXA1, HOXA2, HOXA3, HOXA5, HOXA6, HOXA13, ZNF862, UBE2R2, RAB11FIP2, FBXL20, TUBD1, KPNB1, RPS6KB1, PIGL, FBXW7, SH3D19, HGFAC, C2CD5, COL12A1, EYA4, LMNB1, SEC24A, TXNDC15, CNTN3, COL7A1, ZBTB47, NKTR, IFIH1, GCA, BIRC6, STRN, PLCL1, COX7A2L, SCP2, WRAP73, TBCE, SIPA1L2, PLPPR4, RSRP1, NSL1, SENP5, YIPF4, EPCAM, FAM45A, NUP43, PCMT1, CRISPLD1, ECHDC2, PSPC1, DPPA4, ZMYM2, TBC1D15, KHDRBS1, TMEM39B, HOXA7, SLC25A16, HOXC13, HOXC11, HOXC12, NCOA3, PEPD, CNOT1, GOT2, KTN1, SGPP1, IDUA, FGFRL1, TMEM175, WFIKKN1, WDR24, FBXL16, CHTF18, GNG13, MTUS1, ITFG1, PHF10, C6ORF203, CYP2E1, C16ORF13, UBE4B, LATS1, UBE2G1, PCBD2, ERAL1, DMGDH, ZMYM5, SRRM1, KRBA1, DPH6, WNT2B, TMEM241, RNF138, WDR36, EPHA7, MRPS9, RAC1, UBAP2, DENND4C, HAUS2, RAB1A, ACTR2, TET1, SPPL2A, CPNE8, MDGA2, WDR89, C14ORF79, RHOT2, NCOR1, PRPSAP2, NPEPPS, PTRH2, GAREM1, CAPN10, CELA2A, NPHP1, GPR17, ANKMY1, ALDH1L1, SLIT2, DGKQ, SLC26A1, RPS3A, BHMT, DDX46, SHPRH, SLC22A3, SNTG1, WHSC1L1, NAPRT, PLIN2, GPR107, ANKRD30A, FRA10AC1, MTG1, NKX6-2, JMY, CCDC50, SAR1B, GGPS1, STK32B, TRAPPC8, PTPRD, CEBPG, PPP2R5E, CHST9, SLFN13, SETD9,

MIER3, PSD3, LHFPL4, ATP2B2, DDX19B, ST3GAL2, APPL1, RHPN1, TPRG1L, STARD9, RGS12, CCDC117, ZBTB8A, ZNF208, POLR3K, SNRNP25, PRR35, WDR90, JMJD8, CCDC78, MEIOB, CMPK1, COA7, ZYG11B, PLPP3, GMEB1, LZIC, RBP7, MEGF6, BRINP3, FLVCR1, KIF26B, B3GALNT2, MEMO1, DPY30, ALB, HESX1, LIPH, SENP2, LRPAP1, RAD54L2, TEX264, ABCE1, OTUD4, GRIK2, TXLNB, STXBP5, CAMLG, CSMD3, MICALL2, INTS1, PHAX, ALDH7A1, UBAP1, PHYHIPL, STOX1, C2, CENPV, ATF7IP2, AGBL1, STIM1, RRM1, PRR25, ULK4, ARF4, DDX19A, STXBP6, GRM5, CLIC4, MYRIP, UBB, PYM1, CDCA4, PRKCDBP, KCNG3, ATP6V0E2, COL24A1, SYCE1, CFAP46, TVP23B, FAM195A, PDE3A, SLFN11, HOXC5, ARPP21, FAM222B, KDM2A, RHOD, NMNAT1, GALNTL6, DENND6A, PDE12, LRRC75A-AS1, VCPIP1, MCTP1, MRPL48, RAB6A, ZDHHC21, DOK7, ZBTB8OS, RHOG, TBL1XR1, CASC2, PPP1R42, PARD6G, PLEC, RNF212, FAM220A, MCMDC2, PARP10, GRINA, FAM101A, GAK, PACS2, ABCA13, TIGD5, C12ORF40, FAHD1, PAK2, HOXC9, HOXC10, LRRC57, LRRC75A, ZNF467, ANKRD62, C5ORF24, OR4P4, UNC5C, ZNF804B, SPNS3, TRAK1, MAFA, CRIP2, MTA1, SPNS2, CSMD1, GPR132, DGCR6, CHEK2, NUDT14, PTP4A2, ADRA2C, PKP3, NCMAP, WDR27, KCNH7, BTBD6, JAG2, TMEM121, ROBO2, BRF1, SGCZ, ANO9, C6ORF120, SIGIRR, C14ORF80, ROR1, PRKG1, MYBL1,

								KATNA1, FNBP1, LUZP2, TRPV2, DUSP28, DPYD, RBM34, MSANTD1, FAT4, LCOR, NCOR2, TMEM26, HTT, SSPO, RAB40C, HOXA4, HOXC6, KLHDC1, DNM3, CYP2A7, QRICH1, HOXC4, FLVCR1-AS1, LRBA, RYR2, LRRTM3, CES1, DCAF12, MIR21, MIR371A, MIR372, MIR373, TATDN3, SPRN, ZYG11A, RUFY2, STK19, DXO, SKIV2L, NELFE, MROH6, ATP6V0E2-AS1, PSG5, RNVU1-17, MIR615, MIR661, MIR662, MIR196B, MIR516A2, MIR559, MIR196A2, MIR527, MIR519A1, PTTG3P, CRIP1, ATF6B, C8ORF44, SCART1, CELA2B, MIR1254-1, MIR1236, C4B, TEX22, TP73-AS1, HOTAIR, LINC01205, CYP21A2, SMIM1, RP11-545I5.3, DGCR5, SNORD121B, HOXA11-AS, CEACAMP10, RP11-755B10.3, PSG11, CFB, C4A, MIR4300HG, ALDH1L1-AS2, DPH6-AS1, FGF10-AS1, HOXC13-AS, HOXC-AS2, HOXC-AS1, RP3-513G18.2, HOXC-AS3, AC005355.2, RP11-521B24.3, HOXA10-AS, HOXA10, HOXA-AS3, CYP2A6, NHLRC4, EPPK1, RP11-529K1.2, TCF24, MIR4687, MIR4753, MIR1273F, MIR4655, MIR5697, MIR5708, MIR1273G, MIR5587, MIR5095, MIR3176, CTD-2246P4.1, KCNQ1OT1, C8ORF44-SGK3, DGCR9, ZBTB8B, MIR6765, MIR451B, MIR8071-1, MIR6880, LINC00540, MIR8071-2, SNORD49A, SNORD65, MIR4732, EIF1B-AS1, LINC00254
M00803_1	E2F; motif: GGCGSG	14303	630	449	3.1	71.3	2.86E-05	*RAD52, MAD1L1, HOXA11, RPUSD1, RHBDF1, PIGQ, FYN, TTC27, SPAST, KPNA6, LRRC7, HOXC8, JADE2, CNTLN, KCNQ1, ARID4B, SYNE2, SPO11, FAM168A,*

MTA3, CCAR1, VMP1, DGKA, MTFR1, SYT1, INPP5A, MAST4, CLTCL1, KCNQ2, SEMA3C, HOXA9, TP73, SMARCA2, ERC1, SLCO1A2, P3H2, RAB11FIP3, AARS, ZC3HC1, TRPM7, SNAP23, MFSD11, MSH2, PRODH, CRYBB2P1, HSCB, XBP1, PPP6R2, POLE2, CHGA, ITPK1, RAE1, PLCB4, RNF125, RIOK3, PHKB, NPRL3, MPG, NARFL, HAGHL, FAM173A, METRN, STUB1, RHBDL1, ZNF106, SGK3, ARMC1, TRPS1, PYCRL, EEF1D, TUSC3, HOXA1, HOXA2, HOXA5, HOXA6, HOXA13, UBE2R2, RAB11FIP2, FBXL20, TUBD1, KPNB1, RPS6KB1, PIGL, FBXW7, SH3D19, C2CD5, COL12A1, EYA4, LMNB1, SEC24A, TXNDC15, CNTN3, COL7A1, ZBTB47, NKTR, IFIH1, GCA, BIRC6, STRN, PLCL1, COX7A2L, SCP2, WRAP73, TBCE, SIPA1L2, RSRP1, NSL1, SENP5, YIPF4, EPCAM, FAM45A, NUP43, PCMT1, ECHDC2, PSPC1, DPPA4, ZMYM2, TBC1D15, KHDRBS1, TMEM39B, HOXA7, SLC25A16, HOXC13, HOXC12, NCOA3, CNOT1, GOT2, KTN1, SGPP1, IDUA, FGFRL1, TMEM175, WFIKKN1, WDR24, FBXL16, CHTF18, MTUS1, ITFG1, PHF10, C6ORF203, CYP2E1, C16ORF13, UBE4B, LATS1, UBE2G1, PCBD2, ERAL1, DMGDH, ZMYM5, SRRM1, KRBA1, DPH6, TMEM241, RNF138, EPHA7, MRPS9, RAC1, UBAP2, DENND4C, HAUS2, RAB1A, ACTR2, TET1, DNA2, SPPL2A, CPNE8, MDGA2, WDR89, C14ORF79, RHOT2, NCOR1, PRPSAP2, NPEPPS, PTRH2, GAREM1, CELA2A, ANKMY1, ALDH1L1, SLIT2, DGKQ,

SLC26A1, RPS3A, BHMT, DDX46, SHPRH, SLC22A3, SNTG1, WHSC1L1, NAPRT, PLIN2, GPR107, ANKRD30A, MTG1, NKX6-2, ANK3, JMY, SLC30A6, SAR1B, GGPS1, STK32B, TRAPPC8, PTPRD, CEBPG, CHST9, SLFN13, SETD9, MIER3, PSD3, LHFPL4, ATP2B2, DDX19B, ST3GAL2, APPL1, RHPN1, TPRG1L, STARD9, RGS12, CCDC117, ZBTB8A, ZNF208, POLR3K, SNRNP25, PRR35, WDR90, JMJD8, CCDC78, MEIOB, CMPK1, COA7, ZYG11B, PLPP3, GMEB1, LZIC, RBP7, MEGF6, FLVCR1, KIF26B, B3GALNT2, MEMO1, DPY30, DPPA2, ALB, HESX1, LIPH, SENP2, LRPAP1, RAD54L2, TEX264, OTUD4, STXBP5, CAMLG, CSMD3, MICALL2, INTS1, PHAX, ALDH7A1, UBAP1, PHYHIPL, STOX1, C2, CENPV, ATF7IP2, STIM1, RRM1, PRR25, ULK4, ARF4, DDX19A, STXBP6, GRM5, CLIC4, MYRIP, UBB, PYM1, CDCA4, PRKCDBP, KCNG3, ATP6V0E2, COL24A1, SYCE1, CFAP46, TVP23B, FAM195A, PDE3A, HOXC5, ARPP21, FAM222B, KDM2A, RHOD, NMNAT1, TOMM20, GALNTL6, DENND6A, PDE12, DNAH12, LRRC75A-AS1, VCPIP1, MCTP1, MRPL48, RAB6A, ZDHHC21, DOK7, ZBTB8OS, RHOG, TBL1XR1, CASC2, PPP1R42, PARD6G, PLEC, RNF212, FAM220A, MCMDC2, GRINA, FAM101A, GAK, EDC3, PACS2, ABCA13, TIGD5, C12ORF40, FAHD1, PAK2, HOXC9, HOXC10, LRRC57, LRRC75A, C5ORF24, UNC5C, ZNF804B, SPNS3, TRAK1, MAFA, CRIP2, MTA1, SPNS2, CSMD1, GPR132,

								DGCR6, CHEK2, NUDT14, PTP4A2, ADRA2C, PKP3, NCMAP, WDR27, BTBD6, JAG2, TMEM121, BRF1, SGCZ, ANO9, C6ORF120, SIGIRR, C14ORF80, ROR1, PRKG1, MYBL1, KATNA1, QRFPR, FNBP1, LUZP2, TRPV2, DUSP28, DPYD, RBM34, FAT4, LCOR, NCOR2, TMEM26, HTT, RAB40C, HOXA4, HOXC6, KLHDC1, DNM3, QRICH1, HOXC4, FLVCR1-AS1, LRBA, RYR2, MIR21, MIR371A, MIR372, MIR373, TATDN3, SPRN, ZYG11A, RUFY2, STK19, DXO, SKIV2L, NELFE, ATP6V0E2-AS1, SNORA14B, RNVU1-17, MIR615, MIR661, MIR662, MIR196B, MIR559, MIR196A2, MIR527, CRIP1, ATF6B, C8ORF44, CELA2B, MIR1236, MIR1283-2, TEX22, TP73-AS1, C4B-AS1, AC073283.4, SMIM1, RP11-545I5.3, DGCR5, SNORD121B, SNORD121A, HOXA11-AS, CEACAMP10, RP11-755B10.3, ALDH1L1-AS2, DPH6-AS1, HOXC13-AS, HOXC-AS2, HOXC-AS1, HOXC-AS3, RP11-521B24.3, HOXA10-AS, HOXA10, NHLRC4, EPPK1, RP11-529K1.2, TCF24, MIR4687, MIR1273F, MIR4655, MIR5697, MIR5095, KCNQ1OT1, C8ORF44-SGK3, DGCR9, ZBTB8B, MIR6765, MIR6880, SNORD49A, MIR6817, EIF1B-AS1, LINC00254, SNORA50A
M01177_1	SREBP-2; motif: NNGYCACNNSMN	13504	630	420	3.1	66.7	1.47E-03	*MAD1L1, HOXA11, RPUSD1, RHBDF1, FYN, ZC3H3, PLEKHB1, SPAST, KPNA6, LRRC7, HOXC8, JADE2, KCNQ1, ARID4B, SYNE2, FAM168A, MTA3, CCAR1, VMP1, DGKA, MTFR1, SYT1, FGF10, KCNQ2, TP73, SMARCA2, FYB, P3H2, RAB11FIP3, AARS, NLRC4, TRPM7, MFSD11, MSH2,*

PRODH, CRYBB2P1, POLE2, CHGA, ITPK1, PLCB4, RNF125, RIOK3, FGF14, MSLN, PHKB, SLC38A7, NPRL3, NARFL, HAGHL, FAM173A, METRN, STUB1, RHBDL1, CAPN15, SGK3, ARMC1, GSDMD, PYCRL, EEF1D, HOXA1, HOXA2, HOXA5, HOXA6, HOXA13, ZNF862, UBE2R2, TUBD1, KPNB1, RPS6KB1, PIGL, FBXW7, SH3D19, HGFAC, C2CD5, EYA4, LMNB1, NR3C1, SEC24A, TXNDC15, CNTN3, KNG1, COL7A1, ZBTB47, NKTR, BIRC6, SCP2, TBCE, SIPA1L2, NSL1, SENP5, YIPF4, EPCAM, FAM45A, PCMT1, ECHDC2, PSPC1, DPPA4, ZMYM2, KHDRBS1, TMEM39B, HOXA7, SLC25A16, HOXC13, HOXC11, HOXC12, NCOA3, PEPD, PSG8, CNOT1, GOT2, MED1, SGPP1, IDUA, FGFRL1, WFIKKN1, WDR24, FBXL16, CHTF18, GNG13, MTUS1, ITFG1, PHF10, CYP2E1, C16ORF13, LATS1, UBE2G1, PCBD2, ERAL1, ZMYM5, TMEM241, EPHA7, RAC1, UBAP2, DENND4C, HAUS2, RAB1A, ACTR2, TET1, DNA2, SPPL2A, CPNE8, WDR89, C14ORF79, RHOT2, PRPSAP2, PTRH2, GAREM1, CAPN10, NLRP12, CELA2A, NPHP1, GPR17, ALDH1L1, VPRBP, SLIT2, DGKQ, SLC26A1, DDX46, SLC22A3, SNTG1, WHSC1L1, NAPRT, GPR107, ANKRD30A, FAM13C, MTG1, NKX6-2, PCDH15, JMY, OLAH, SLC30A6, SAR1B, GGPS1, STK32B, TRAPPC8, CEBPG, PPP2R5E, CHST9, MIER3, PSD3, LHFPL4, ATP2B2, DDX19B, APPL1, RHPN1, TPRG1L, RGS12, CCDC117, ZBTB8A, POLR3K, SNRNP25, PRR35,

WDR90, JMJD8, CCDC78, MEIOB, COA7, ZYG11B, PLPP3, GMEB1, LZIC, RBP7, MEGF6, CCDC27, BRINP3, FLVCR1, KIF26B, B3GALNT2, MEMO1, DPY30, DPPA2, HESX1, SENP2, LRPAP1, RAD54L2, TEX264, ABCE1, OTUD4, PAPD4, STXBP5, CAMLG, MICALL2, UBAP1, STOX1, C2, CENPV, ATF7IP2, AGBL1, STIM1, RRM1, PRR25, ULK4, ARF4, DDX19A, STXBP6, MYO7B, MYRIP, UBB, CDCA4, PSG6, PRKCDBP, KCNG3, ATP6V0E2, COL24A1, SYCE1, CFAP46, TVP23B, SLFN12, FAM195A, HOXC5, ARPP21, KDM2A, RHOD, NMNAT1, TOMM20, DNAH12, LRRC75A-AS1, VCPIP1, MCTP1, MRPL48, RAB6A, DOK7, ZBTB8OS, RHOG, PPP1R42, PARD6G, PLEC, FAM220A, MCMDC2, PARP10, GRINA, FAM101A, EDC3, PACS2, TIGD5, FAHD1, PAK2, HOXC10, LRRC57, ZNF467, ANKRD62, C5ORF24, UNC5C, TRAK1, MAFA, CRIP2, MTA1, CSMD1, GPR132, DGCR6, NUDT14, PTP4A2, ADRA2C, PKP3, NCMAP, KCNH7, BTBD6, JAG2, TMEM121, BRF1, SIGIRR, C14ORF80, ROR1, SPATA45, PRKG1, KATNA1, QRFPR, RHD, FNBP1, TRPV2, SLC24A5, RBM34, MSANTD1, LCOR, NCOR2, TEX43, TMEM26, HTT, SSPO, RAB40C, HOXA4, HOXC6, DNM3, CYP2A7, QRICH1, HOXC4, FLVCR1-AS1, LRBA, CES1, MIR21, MIR371A, MIR372, MIR373, TATDN3, SPRN, ZYG11A, RUFY2, STK19, DXO, MROH6, ATP6V0E2-AS1, PSG5, SNORA14B, RNVU1-17, MIR615, MIR661, MIR662, MIR196B, MIR516A2, MIR551A,

							MIR559, MIR196A2, MIR527, MIR519A1, PTTG3P, CRIP1, C8ORF44, RP11-1023L17.2, CELA2B, MIR1288, MIR1283-2, C4B, LINC01087, TEX22, TP73-AS1, HOTAIR, LINC01205, C4B-AS1, CYP21A2, SMIM1, LINC00993, CDKN2AIPNL, DGCR5, SNORD121B, MIR2053, SNORD121A, ASB14, HOXA11-AS, CEACAMP10, PSG2, PSG11, C4A, ALDH1L1-AS2, FGF10-AS1, HOXC13-AS, RP3-513G18.2, HOXC-AS3, AC005355.2, RP11-521B24.3, HOXA10-AS, HOXA10, CYP2A6, NHLRC4, EPPK1, RP11-529K1.2, MIR4687, MIR4753, MIR1273F, MIR4655, MIR5697, MIR5708, MIR1273G, MIR5587, MIR5585, MIR3176, KCNQ1OT1, C8ORF44-SGK3, DGCR9, ZBTB8B, MIR6765, MIR451B, MIR8071-1, MIR6880, MIR8071-2, SNORD49A, SNORD65, MIR4732, EIF1B-AS1, LINC00254, SNORA50A
M07231_0	TFAP2A; motif: NNNNGCCYSAGGGCA	2513	630	106	4.2	16.8	2.38E-03	*RAD52, RPUSD1, PIGQ, ZC3H3, HOXC8, KCNQ1, SPO11, KCNQ2, ERC1, CAPN15, GSDMD, PYCRL, HOXA3, HOXA5, KPNB1, HGFAC, EYA4, KNG1, COL7A1, SCP2, RSRP1, YIPF4, CRISPLD1, ZMYM2, KTN1, FGFRL1, WDR24, CHTF18, C16ORF13, ZMYM5, DENND4C, CPNE8, RHOT2, ANKMY1, DGKQ, SLC26A1, MTG1, SLC30A6, PSD3, ATP2B2, ST3GAL2, RGS12, WDR90, JMJD8, CMPK1, LZIC, MEGF6, MEMO1, LRPAP1, TXLNB, CSMD3, INTS1, PHYHIPL, PRR25, CLIC4, CDCA4, PSG6, PRKCDBP, SYCE1, FAM195A, PDE3A, FAM222B, NMNAT1, ZDHHC21, DOK7, PPP1R42, PLEC, PARP10, LRRC75A,*

									ZNF467, PSG9, NUDT14, ADRA2C, NCMAP, TMEM121, C14ORF80, RHD, TRPV2, DUSP28, MSANTD1, SSPO, CYP2A7, CES1, DCAF12, ZYG11A, PSG5, MIR661, MIR662, CRIP1, C4B, LINC01087, PSG1, SMIM1, PSG2, PSG11, PSG4, C4A, LINC00536, RP11-215P8.4, RP11-521B24.3, HOXA-AS3, NHLRC4, MIR1273F, MIR4655, MIR3176, MIR6765
	M01588_1	GKLF; motif: GCCMCRCCCNNN	183	630	18	9.8	2.9	3.79E-03	*RPUSD1, PIGQ, FAM173A, STRN, NCOA3, CHTF18, CYP2E1, PRPSAP2, DPPA2, PACS2, CRIP2, SPNS2, NUDT14, PTP4A2, C14ORF80, MIR661, RP3-513G18.2, NHLRC4*
	M06621_1	ZNF479; motif: NSKACAAAMAGC	17	630	6	35.3	1	5.78E-03	*PIGQ, PYCRL, SCP2, C14ORF79, PARP10, NHLRC4*
CNVRs only in the placental genomes of miscarried RPL pregnancies									
GO	0050911	detection of chemical stimulus involved in sensory perception of smell	408	110	19	4.7	17.3	3.84E-09	*OR7G1, OR7G3, OR7G2, OR1M1, OR2T1, OR2T35, OR2T29, OR2T11, OR2T34, OR2T10, OR2T27, OR7D2, OR2G6, OR14I1, OR2T2, OR2T3, OR2T4, OR2T6, OR2T5*
	0004984	olfactory receptor activity	408	110	19	4.7	17.3	3.84E-09	*OR7G1, OR7G3, OR7G2, OR1M1, OR2T1, OR2T35, OR2T29, OR2T11, OR2T34, OR2T10, OR2T27, OR7D2, OR2G6, OR14I1, OR2T2, OR2T3, OR2T4, OR2T6, OR2T5*
	0004930	G-protein coupled receptor activity	867	110	19	2.2	17.3	9.55E-04	*OR7G1, OR7G3, OR7G2, OR1M1, OR2T1, OR2T35, OR2T29, OR2T11, OR2T34, OR2T10, OR2T27, OR7D2, OR2G6, OR14I1, OR2T2, OR2T3, OR2T4, OR2T6, OR2T5*

Supplementary Table S4. Summary of FPKM values for 'CNV genes' compared to 'non CNV genes'. See details for the definitions in Methods.

**ALL SAMPLES**							
**Gene group**	**genes (n)**	**Min.**	**1st Quartile**	**Median**	**Mean**	**3rd Quartile**	**Max.**
nonCNV	45,156	0	0	0.0938	4.37	1.27	13460
CNV	2,273	0	0	0.1511	4.42	2.08	340.4
del+dupl	62	0	0	0.0661	23.71	1.06	340.4
del	1,148	0	0	0.3413	4.71	3.55	306.7
dupl	1,063	0	0	0.0489	2.98	0.96	277
**TERM PLACENTA**							
**Gene group**	**genes (n)**	**Min.**	**1st Quartile**	**Median**	**Mean**	**3rd Quartile**	**Max.**
nonCNV	45,266	0	0	0.0946	4.32	1.25	13060
CNV	2,163	0	0	0.1523	4.46	2.16	356.8
del+dupl	41	0	0	0.0350	34.99	1.49	340.4
del	1,112	0	0	0.3707	4.63	3.69	356.8
dupl	1,010	0	0	0.0479	3.02	0.95	290.9

Note:

Analysis for the effect of CNVs on the expression of the involved genes was conducted in parallel for all placental samples with available RNA-Seq data[3,4] (n=50) and separately in term pregnancy samples[3] (n=40) only. The analysis was restricted to the genes having concordant id in Ensembl v67 and Ensembl v87 (n=47,429). Start and end coordinates (hg38) of CNV regions (n=1,257 across all samples; n=1094 in term samples) were compared to gene coordinates from Ensembl v87. Genes with any overlap with CNV regions were classified as 'CNV genes' (n=2,273 in all samples; n=2,163 in term samples). 'CNV genes' were subdivided as genes present only in deletion CNVs (del; n=1,148 in all samples; n=1,112 in term samples), present only in duplication CNVs (dupl; n=1,063 all samples; n=1,010 term samples). Genes detected in both deletion and duplication CNVs (n=62 all samples; n=41 term samples) were not analysed separately due to their small number. Median FPKM (fragments per kilobase per million) values in all samples and term samples were extracted for each gene.

Supplementary Table S5. Copy number variants identified in RPL family trios with a term live birth after a successful pregnancy. Genomic coordinates are given according to hg38. The analysis included only autosomal CNVs. Chr, chromosome; Del, deletion, Dupl, duplication; n.a., not applicable. Large pericentromeric/subtelomeric CNVs are shown in **bold font**.

Chr	Start (hg38)	End	Length	Sample	Type	Inherited/somatic in the placental genome
1	103611243	103613322	2,079	RPL11 placenta	Del	somatic
1	162762701	162769219	6,518	RPL11 mother	Del	n.a.
1	231573658	231677388	103,730	RPL11 father	Dupl	n.a.
1	238929939	238932730	2,791	RPL11 placenta	Del	somatic
2	57172695	57218336	45,641	RPL11 father	Dupl	n.a.
2	212322309	212325270	2,961	RPL11 father	Del	n.a.
2	241975583	242106609	131,026	RPL11 mother	Del	n.a.
3	19817	90294	70,477	RPL11 mother	Dupl	n.a.
3	1515729	1680597	164,868	RPL11 mother	Dupl	n.a.
4	164655588	164665848	10,260	RPL11 placenta	Del	somatic
5	788531	840602	52,071	RPL11 mother	Del	n.a.
6	137578655	137644281	65,626	RPL11 placenta	Dupl	somatic
**7**	**55274956**	**55838203**	**563,247**	**RPL11 father**	**Del**	**n.a.**
8	5743669	5747565	3,896	RPL11 mother	Del	n.a.
9	7887951	7891408	3,457	RPL11 mother	Del	n.a.
10	118826403	119033191	206,788	RPL11 placenta	Dupl	somatic
**13**	**18484577**	**18943280**	**458,703**	**RPL11 mother**	**Dupl**	**n.a.**
13	95284895	95380685	95,790	RPL11 father	Dupl	n.a.
14	23960040	24014851	54,811	RPL11 mother	Del	n.a.
**14**	**105581568**	**105884536**	**302,968**	**RPL11 mother**	**Dupl**	**n.a.**
15	24152472	24170857	18,385	RPL11 mother	Del	n.a.
15	57352258	57366684	14,426	RPL11 placenta	Del	somatic
16	81190041	81267966	77,925	RPL11 mother	Del	n.a.
17	46092442	46272724	180,282	RPL11 father	Dupl	n.a.
19	50757732	50760253	2,521	RPL11 placenta	Dupl	somatic
21	15584547	15937614	353,067	RPL11 mother	Dupl	n.a.
21	30720114	30866332	146218	RPL11 mother	Dupl	n.a.
1	10612878	10679198	66,320	RPL12 placenta	Dupl	somatic
1	70542243	70553303	11,060	RPL12 father	Del	n.a.
2	109720215	110222769	502,554	RPL12 placenta	Del	somatic
2	183933099	183937882	4,783	RPL12 placenta	Del	somatic
2	241931649	242106609	174,960	RPL12 father	Del	n.a.
3	19817	102299	82,482	RPL12 father	Dupl	n.a.
3	143447087	143476613	29,526	RPL12 father	Del	n.a.
3	159558237	159580247	22,010	RPL12 mother	Del	n.a.
3	176175294	176189554	14,260	RPL12 father	Del	n.a.

4	2248382	2259993	11,611	RPL12 placenta	Dupl	somatic
4	3720680	3873507	152,827	RPL12 placenta	Dupl	somatic
4	93518875	93713151	194,276	RPL12 father	Del	n.a.
5	8707907	8744445	36,538	RPL12 placenta	Del	somatic
5	131681245	131740228	58,983	RPL12 placenta	Del	somatic
8	103411258	103416302	5,044	RPL12 mother	Del	n.a.
9	133204076	133210396	6,320	RPL12 mother	Del	n.a.
9	134393077	134411093	18,016	RPL12 placenta	Dupl	somatic
9	136606165	136624513	18,348	RPL12 placenta	Dupl	somatic
10	89184459	89185999	1,540	RPL12 placenta	Del	somatic
12	31848581	31919612	71,031	RPL12 father	Dupl	n.a.
16	80857994	80910771	52,777	RPL12 placenta	Del	somatic
17	46092442	46272724	180,282	RPL12 mother	Dupl	n.a.
19	15653506	15654151	645	RPL12 mother	Del	n.a.
19	40850846	40873243	22,397	RPL12 placenta	Del	somatic
1	3451579	3734754	283,175	RPL3 placenta	Dupl	somatic
2	238116989	238167171	50,182	RPL3 father	Dupl	n.a.
5	7178531	7190961	12,430	RPL3 mother	Del	n.a.
5	97712762	97763616	50,854	RPL3 father	Del	n.a.
5	97712762	97781411	68,649	RPL3 placenta	Del	inherited
6	31253262	31254137	875	RPL3 father	Del	n.a.
7	111252170	111383266	131,096	RPL3 father	Del	n.a.
7	111252170	111385477	133,307	RPL3 placenta	Del	inherited
8	5741877	5747565	5,688	RPL3 father	Del	n.a.
9	126886252	126897639	11,387	RPL3 placenta	Del	somatic
16	21305426	21828019	522,593	RPL3 father	Del	n.a.
16	21305426	21828019	522,593	RPL3 placenta	Del	inherited
16	76886122	76981412	95,290	RPL3 mother	Del	n.a.
**19**	**27428816**	**27752973**	**324,157**	**RPL3 father**	**Dupl**	**n.a.**
19	46294573	46313531	18,958	RPL3 father	Del	n.a.
20	15776678	15783700	7,022	RPL3 mother	Del	n.a.

References to Supplementary Materials

1. Nagirnaja, L. *et al.* Structural Genomic Variation as Risk Factor for Idiopathic Recurrent Miscarriage. *Hum. Mutat.* **35**, 972-82 (2014)**.**

2. Kasak, L., Rull, K., Vaas, P., Teesalu, P. & Laan, M. Extensive load of somatic CNVs in the human placenta. *Sci. Rep.* **5**, 8342 (2015).

3. Sõber, S. *et al.* Extensive shift in placental transcriptome profile in preeclampsia and placental origin of adverse pregnancy outcomes. *Sci. Rep.* **5**, 13336 (2015).

4. Sõber, S. *et al.* RNA sequencing of chorionic villi from recurrent pregnancy loss patients reveals impaired function of basic nuclear and cellular machinery. *Sci. Rep.* **6**, 38439 (2016).

5. Reimand, J., Kull, M., Peterson, H., Hansen, J. & Vilo, J. g:Profiler--a web-based toolset for functional profiling of gene lists from large-scale experiments. *Nucleic Acids Res.* **35**, W193-200 (2007).
